# Bridging the gap: meta-epidemiological analysis on the clinical translation of stem cell-based therapies in women’s reproductive diseases

**DOI:** 10.1093/hropen/hoag024

**Published:** 2026-03-21

**Authors:** Hankun Su, Yu Jian, Ronghui Tang, Hoksan Chau, Xinyu Cheng, Xiao Liu, Yuqian Tong, Jinyao Ning, Xinhua Zhang, Jiayi Chen, Yilin Zhang, Zixin Tong, Yuemeng Yang, Yunyang Zhao, Liye Sun, Jingjing Chen, Hui Li

**Affiliations:** Department of Reproductive Medicine, Xiangya Hospital, Central South University, Changsha, China; Clinical Research Center for Women’s Reproductive Health in Hunan Province, Xiangya Hospital, Central South University, Changsha, China; Department of Reproductive Medicine, Xiangya Hospital, Central South University, Changsha, China; Clinical Research Center for Women’s Reproductive Health in Hunan Province, Xiangya Hospital, Central South University, Changsha, China; Department of Fetal Medicine, Xiangya Hospital, Central South University, Changsha, China; Department of Obstetrics and Gynecology, Hunan University of Medicine General Hospital, Huaihua, China; Department of Reproductive Medicine, Xiangya Hospital, Central South University, Changsha, China; Clinical Research Center for Women’s Reproductive Health in Hunan Province, Xiangya Hospital, Central South University, Changsha, China; Department of Reproductive Medicine, Xiangya Hospital, Central South University, Changsha, China; Clinical Research Center for Women’s Reproductive Health in Hunan Province, Xiangya Hospital, Central South University, Changsha, China; Department of Reproductive Medicine, Xiangya Hospital, Central South University, Changsha, China; Clinical Research Center for Women’s Reproductive Health in Hunan Province, Xiangya Hospital, Central South University, Changsha, China; Department of Reproductive Medicine, Xiangya Hospital, Central South University, Changsha, China; Clinical Research Center for Women’s Reproductive Health in Hunan Province, Xiangya Hospital, Central South University, Changsha, China; Department of Reproductive Medicine, Xiangya Hospital, Central South University, Changsha, China; Clinical Research Center for Women’s Reproductive Health in Hunan Province, Xiangya Hospital, Central South University, Changsha, China; Department of Reproductive Medicine, Xiangya Hospital, Central South University, Changsha, China; Clinical Research Center for Women’s Reproductive Health in Hunan Province, Xiangya Hospital, Central South University, Changsha, China; Department of Reproductive Medicine, Xiangya Hospital, Central South University, Changsha, China; Clinical Research Center for Women’s Reproductive Health in Hunan Province, Xiangya Hospital, Central South University, Changsha, China; Department of Reproductive Medicine, Xiangya Hospital, Central South University, Changsha, China; Clinical Research Center for Women’s Reproductive Health in Hunan Province, Xiangya Hospital, Central South University, Changsha, China; Department of Reproductive Medicine, Xiangya Hospital, Central South University, Changsha, China; Clinical Research Center for Women’s Reproductive Health in Hunan Province, Xiangya Hospital, Central South University, Changsha, China; Department of Reproductive Medicine, Xiangya Hospital, Central South University, Changsha, China; Clinical Research Center for Women’s Reproductive Health in Hunan Province, Xiangya Hospital, Central South University, Changsha, China; Department of Reproductive Medicine, Xiangya Hospital, Central South University, Changsha, China; Clinical Research Center for Women’s Reproductive Health in Hunan Province, Xiangya Hospital, Central South University, Changsha, China; Department of Reproductive Medicine, Xiangya Hospital, Central South University, Changsha, China; Clinical Research Center for Women’s Reproductive Health in Hunan Province, Xiangya Hospital, Central South University, Changsha, China; Department of Reproductive Medicine, Xiangya Hospital, Central South University, Changsha, China; Clinical Research Center for Women’s Reproductive Health in Hunan Province, Xiangya Hospital, Central South University, Changsha, China; Department of Reproductive Medicine, Xiangya Hospital, Central South University, Changsha, China; Clinical Research Center for Women’s Reproductive Health in Hunan Province, Xiangya Hospital, Central South University, Changsha, China

**Keywords:** women’s reproductive diseases, premature ovarian failure, stem cell, exosome, meta-epidemiological study, clinical trial

## Abstract

**STUDY QUESTION:**

What is the current landscape of randomized controlled trials (RCTs) evaluating stem cell-based therapies for women’s reproductive diseases, and how effectively has preclinical research informed their clinical translation?

**SUMMARY ANSWER:**

The current clinical trial landscape for stem cell-based therapies in women’s reproductive diseases is characterized by rapid growth in trial registration, particularly for premature ovarian failure (POF) and intrauterine adhesions (IUA), yet remains predominantly in early-phase, single-center, single-country studies with significant heterogeneity in design and outcome reporting.

**WHAT IS KNOWN ALREADY:**

Stem cell-based therapies show promise in preclinical models for conditions such as POF, IUA, and endometriosis. However, despite increasing clinical interest, the extent to which these therapies have been rigorously evaluated in RCTs and how well clinical trials reflect preclinical evidence remains unclear.

**STUDY DESIGN, SIZE, DURATION:**

This translational analysis integrated a meta-epidemiological approach with bibliometric methods. We searched the International Clinical Trials Registry Platform (ICTRP), ClinicalTrials.gov, EudraCT, and ChiCTR from inception to 1 December 2025, using terms related to ‘stem cells’, ‘reproductive diseases’, and ‘randomized controlled trials’. Only English and Chinese registration records were included.

**PARTICIPANTS/MATERIALS, SETTING, METHODS:**

Two reviewers independently screened registration records for RCTs assessing stem cell or derivative therapies in women’s reproductive diseases. Eligible criteria included RCTs that reported the use of stem cells or their derivatives in female patients with reproductive conditions. Data on trial design, disease focus, stem cell type, source, phase, sample size, outcomes, and results were extracted. A hybrid approach combined traditional systematic data extraction with machine learning-assisted screening (ASReview) for basic research. Bibliometric analysis using VoSviewer and CiteSpace mapped research trends, while Health Research Classification System (HRCS) scores assessed translational potential. Consistency in screening and extraction was evaluated using kappa and Cronbach’s alpha.

**MAIN RESULTS AND THE ROLE OF CHANCE:**

From 804 records, 38 RCTs met inclusion criteria. The majority were early-phase (Phase 1/2, 76.3%), single-center (81.6%), and conducted in one country (97.4%), with China (50.0%) and the USA (18.9%) leading. POF (34.2%), ovarian cancer (23.7%), and IUA (18.4%) were the most studied conditions. Umbilical cord-derived mesenchymal stem cells (28.9%) were the most commonly used. While preclinical research shows strong mechanistic support, especially for POF and IUA, there was minimal overlap in primary outcome measures across trials, limiting comparability. Only 36.8% of RCTs included safety as a co-primary endpoint, and 49% had short-term follow-up, raising concerns about long-term risks such as tumorigenicity.

**LIMITATIONS, REASONS FOR CAUTION:**

The analysis may be subject to language bias (English and Chinese only), and some included trials were incomplete, relying on interim reports or press releases not peer-reviewed. The quality of individual RCTs was not formally assessed, and publication bias in basic research may influence bibliometric trends.

**WIDER IMPLICATIONS OF THE FINDINGS:**

This study highlights a significant gap between robust preclinical evidence and fragmented clinical evaluation, with over-concentration on POF and underrepresentation of other high-potential conditions like endometriosis and thin endometrium. The findings underscore the need for international collaboration, standardized outcome sets, and harmonized regulatory frameworks to enable large-scale, high-quality trials that can translate regenerative potential into clinical reality.

**STUDY FUNDING/COMPETING INTEREST(S):**

This study was supported by the National Natural Science Foundation of China (82301835 and 82371682), the Science and Technology Program of Hunan Province (2024JJ4091 and 2023JJ40956), the Youth Fund of Xiangya Hospital, Central South University, China (2021Q03 and 2209090550121), the Postdoctoral Science Foundation of China (2022M713522 and 2021TQ0372), and the Innovation and Entrepreneurship Training Program for College Students (202510533012, S202410533307, and CXPY2025224). The authors declare no competing interests.

**REGISTRATION NUMBER:**

https://doi.org/10.17605/OSF.IO/JM9EK.

WHAT DOES THIS MEAN FOR PATIENTS? Many women around the world struggle with reproductive health conditions such as early menopause, damage inside the uterus, or infertility, and current treatments often do not fully restore function or offer long-term solutions. In recent years, scientists have been exploring a new kind of treatment using stem cells—special cells that can help repair damaged tissues and improve organ function. These therapies have shown promise in early studies, but it’s not clear how much progress has been made in turning these ideas into proven treatments that can be widely used.

This study looked at all the high-quality clinical trials (studies that test treatments in people) that have been done so far on stem cell therapies for women’s reproductive conditions. We wanted to understand what has been tested, how well the trials were designed, and whether the results from earlier laboratory studies have helped guide these human trials.

We found that while there has been a steady increase in the number of trials over the past two decades—especially for conditions like early menopause and uterine scarring—most are still in the early stages, involve only a small number of patients, and are run in just one country. There is also a lot of variation in how the treatments are given, what kind of stem cells are used, and how success is measured, making it hard to compare results or know which approach works best. Safety is another concern: although stem cells may carry long-term risks such as unwanted tissue growth, many trials do not follow patients for long enough to detect these issues.

On a positive note, the research shows that stem cell treatments could be especially helpful for certain conditions, and new approaches—such as using tiny particles released by stem cells (called exosomes) or combining stem cells with supportive materials like collagen—are emerging as safer and more effective options.

Our findings highlight the need for larger, better-coordinated international studies, clearer standards for how treatments are prepared and tested, and more focus on conditions beyond the most commonly studied ones. By improving how research is done, we can move closer to offering safe and effective regenerative treatments for more women in need.

## Introduction

Women’s reproductive health is fundamental to global well-being, yet conditions like primary ovarian failure (POF), intrauterine adhesions (IUA), endometriosis, and uterine factor infertility impose significant burdens (Sanne *et al.*, 2016; Li *et al.*, 2023b). Current therapies often provide only symptomatic relief, failing to address underlying tissue damage and exhibiting limited long-term efficacy (Becker *et al.*, 2022).

Stem cells and their derivatives have emerged as promising regenerative candidates for these disorders (Liu *et al.*, 2023). Their unique capacities for self-renewal, multi-lineage differentiation, and paracrine signaling offer potential mechanisms for restoring function in conditions like endometriosis, IUA, and POF (Song *et al.*, 2021; Cevik *et al.*, 2025). Currently, research exploring stem cell-based applications in female reproductive health has yielded substantial preclinical data and early-phase clinical evidence, including several randomized controlled trials (RCTs), which is considered as the gold standard for evaluating the effectiveness of medical interventions (Hariton and Locascio, 2018).

Despite this progress, the translation of stem cell-based therapy from bench to bedside remains a significant challenge, with only a few therapies approved for clinical use. Numerous systematic reviews and meta-analyses have previously focused on specific aspects of stem cell therapy in reproductive medicine, such as its role in POF or IUA, often emphasizing clinical outcomes or mechanistic pathways (Shareghi-Oskoue *et al.*, 2021; Hu *et al.*, 2024; Luo *et al.*, 2024; Umer *et al.*, 2024; Yuan *et al.*, 2025). While valuable, these reviews typically provide narrow perspectives and do not comprehensively address the broader translational landscape, including how findings from basic science inform clinical trial design or where future research efforts should be directed.

To address these gaps, this translational analysis aims to examine the current landscape of stem cell-based therapy and identify gaps between bench and bedside. However, traditional systematic reviews offer limited perspectives and struggle to capture the exponentially growing complexity of bench-to-bedside translation (Su *et al.*, 2025). To address this, we integrated bibliometric analysis—a quantitative and visualized method for evaluating the research landscape and trends within the translational analysis (Ninkov *et al.*, 2022). This hybrid approach enables a dual perspective: the bibliometric analysis serves as an indicator, mapping the basic research landscape and offering insights into translational trend, while the translational analysis digs deeper into synthesizing qualitative and quantitative insights in clinical trials. Together, the hybrid approach provides a holistic view of the state of translation regarding to stem cell-based therapy. To systematically address our objectives, we focus on two translational research questions:

RQ.01: What is the current landscape of RCTs for stem cell-based therapies in women’s reproductive health?

RQ.02: How effectively have preclinical findings informed clinical trial design and implementation?

By answering such questions, this study aims to provide a comprehensive overview of the existing RCTs on stem cell-based therapy for women’s reproductive diseases, offering valuable insights into the current state of bench-to-bedside translation and guiding future directions for clinical applications and investigations.

## Methods

This translational analysis was conducted according to the JBI Manual for evidence synthesis and adheres to the guidelines for reporting meta-epidemiological methodology research (Murad and Wang, 2017). This analysis was preregistered at the OSF platform: https://doi.org/10.17605/OSF.IO/JM9EK

### Ethics

This analysis is based on previously published studies; no ethical approval or informed consent was required.

### Search strategy and study selection for RCTs

The International Clinical Trial Registry Platform (ICTRP), Clinicaltrials.gov, EudraCT, and the ChiCTR database were searched as the source of relevant RCTs. Web of Science (WoS) Core Collection and PubMed were searched as supplementary sources for any potentially unregistered RCTs. The search was conducted on 1 December 2025. The detailed search strategy can be found in Supplementary File S1. We used search terms such as: (‘stem cells’ OR ‘mother cells’ OR ‘Progenitor Cells’) AND (‘Reproductive disease’ OR ‘Reproductive diseases’ OR ‘Gynecologic Diseases’ OR ‘Gynecologic Disease’) AND (‘randomized control trials’). Two authors (H.C. and Yi.Z.) screened potentially relevant RCTs. At any point in the process, discrepancies in RCT selection were resolved by senior researcher H.S. The eligible criteria were listed below.

Inclusion criteria:

1. RCTs that focus on the use of stem cell and its derivatives in treating reproductive diseases.
2. Articles that reported outcomes of an RCT related to stem cell-based therapy without any trial registration.

### Machine learning enhanced search selection for basic research

WoS Core Collection and PubMed were searched as the source of relevant articles. The search was first conducted on 7 June 2025. Supplementary File S1 provides detailed search strategies.

For the initial screening phase, Active learning for Systematic Reviews (ASReview version 0.19.2), a machine learning-based app, was used for the screening phase of the reviews and the recommended default settings were used (classifier: Naïve Bayes; Query strategy max; Feature extraction: Term Frequency-Inverse Document Frequency) (van de Schoot *et al.*, 2021). A pilot selection of 50 articles were tested to show consistency between ASReview and a human reviewer (kappa value = 0.896, Supplementary Table S1).

After the first round of initial screening, data were imported into another platform, Rayyan (Ouzzani *et al.*, 2016), a web-based platform for collaborative systematic review screening, where four authors (J.N., X.L., Y.Y., Z.T.) independently performed full-text screening in blinded mode to assess eligibility. At any point in the process, discrepancies in article selection were resolved by senior researcher HK. The eligible criteria were listed below.

Inclusion criteria:

1. Peer-reviewed original research articles that investigate fundamental biological mechanisms, cellular or molecular pathways, or preclinical therapeutic potential of stem cells or their derivatives in the context of women’s reproductive diseases.

Exclusion criteria:

1. Articles that only report derivatives from cell source other than stem cell.
2. Articles reporting results from human clinical trials, including RCTs, case series, or observational studies in patients.
3. Reviews, letters, abstract only.

### Data extraction

Five authors (H.C., X.C., Yi.Z., X.Z., Jia.C.) independently extracted clinical trial-related data using preset templates. For each included record, the following characteristics were extracted: trial registration number, registered date, sponsor, treating diseases, country, centers, sample size, primary outcome, primary result, stem cell type, stem cell source, phase, and reference. A pilot extraction on 20% of trials were used to train and assess the consistency between authors (Cronbach’s alpha = 0.968).

Another four authors (J.N., X.L., Y.Y., Z.T.) independently extracted data from 200 randomly selected basic research articles as representatives for overall basic research. For each included basic research article, the following characteristics were extracted: title, keywords, related disease, and health research classification system (HRCS) score. HRCS score is a novel metric which were used as an indicator of translational versus basic research (Viney, 2013; Hourigan *et al.*, 2025). Generally, it categorized studies into five stages: Stage 1: Investigates normal functions and processes. Stage 2: Understands the processes of a disease as well as the antecedent factors. Stage 3: Identifies new biomarkers and develops new technologies for detection and population screening. Stage 4: Conducts experimental medicine and testing in model systems or experimental settings. Stage 5: Late-stage translational research that focuses on human testing and real-world settings. A pilot extraction on 5% of articles were used to train and assess the consistency between authors (Cronbach’s alpha = 0.725).

### Bibliometric analysis and statistics

The raw data downloaded from WoS and PubMed were first imported into Microsoft Excel 2019 for preliminary collation. Any discrepancies were taken for re-assessment by a senior researcher (H.S.). Bibliometric parameters were exported to VoSviewer (Leiden University, Leiden, The Netherlands) and Citespace (Version 6.2 R2) were used to conduct a keyword clustering analysis and development frontiers analysis of the results (Chen, 2006). The threshold for keyword occurrence was set at 5.

Although VoSviewer effectively displays the co-occurrence status of keywords, it presents limitations in illustrating the difference in keyword occurrence between clinical trials and basic research. Therefore, we utilized a Python program that extracts and compares the occurrence of keywords between basic research articles and clinical trials. Keywords were extracted from clinical trial studies and basic research studies into Microsoft Excel 2019 as input for this analysis. The Python program identifies similar keywords, and assesses differences in their occurrence frequencies between the two study types by using the chi-square test. For low expected counts (e.g. rare keywords) that violate chi-square assumptions, Fisher’s exact test is used. Keywords with a *P*-value < 0.05 and a fold change > 2 were considered to have statistically significant differences in prevalence.

SPSS 25 (Statistical Product Service Solutions 25; IBM Corporation, Armonk, NY, USA) was adopted to calculate kappa values and Cronbach’s alpha values. GraphPad Prism 9 (GraphPad Software Inc., CA, USA) was used to draw graphs.

## Results

### RCT selection and characteristics

**Figure 1.**
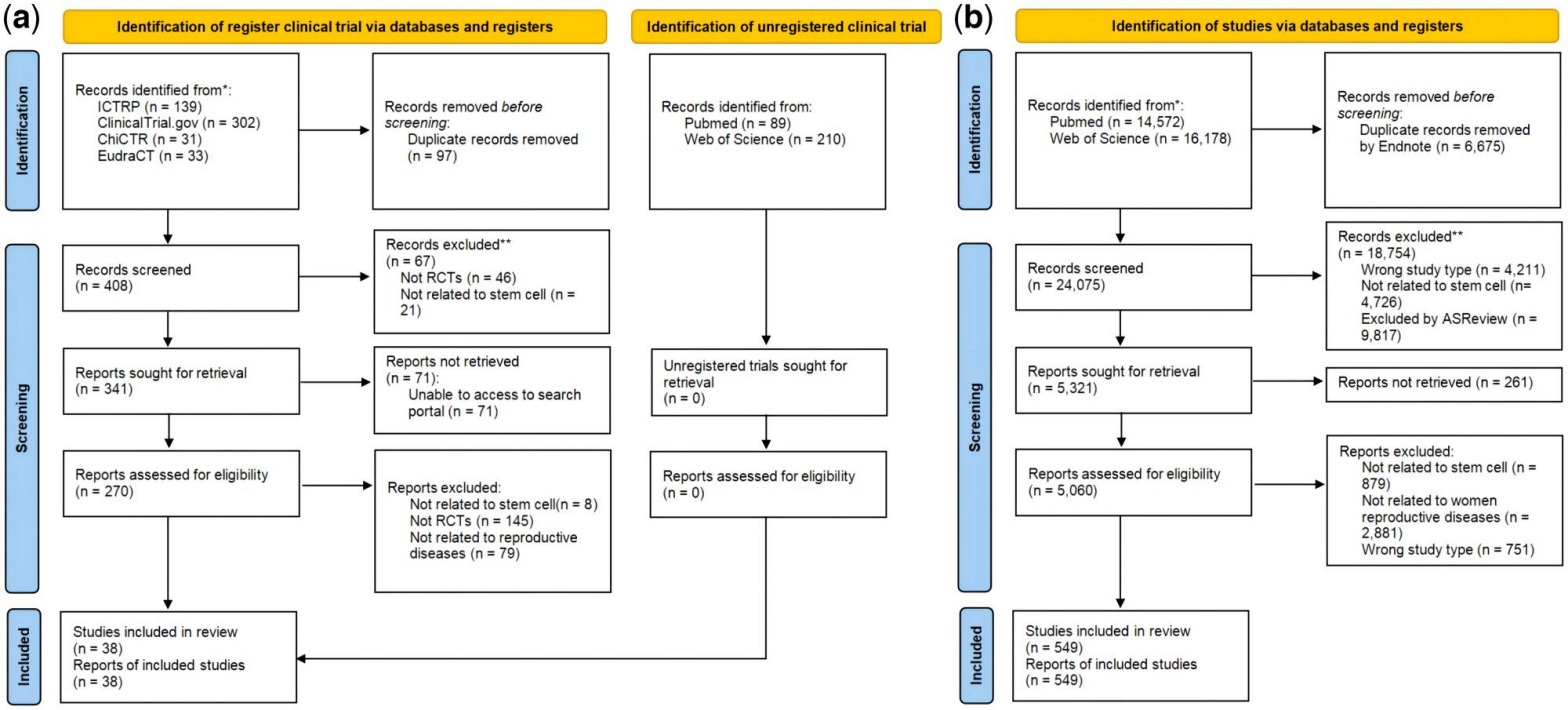
**Illustration of study selection process.** (**a**) Study selection process of RCTs; (**b**) Study selection process of basic research. RCT, randomized controlled trials.

Figure 1a displays the RCT selection process. The electronic search retrieved 804 trial records from Web of Science, PubMed, ICTRP, Clinicaltrials.gov, EudraCT, and ChiCTR platform databases. Duplicates were first removed and after title and abstract screening, 270 trial records were retained for full-text review, resulting in a total of 38 RCTs meeting inclusion criteria. The references and characteristics for all the included RCTs are available in Supplementary Table S2.

Of 38 RCTs, nine disease domains were involved. Thirteen RCTs (34.2%) were related to POF, nine (23.7%) to ovarian cancer, and seven (18.4%) to IUA. Sclnow Biotechnology Co., Ltd. is a major commercial sponsor, initiating three RCTs, primarily focused on thin endometrium (2 RCTs) and premature ovarian failure (1 RCT).

**Figure 2.**
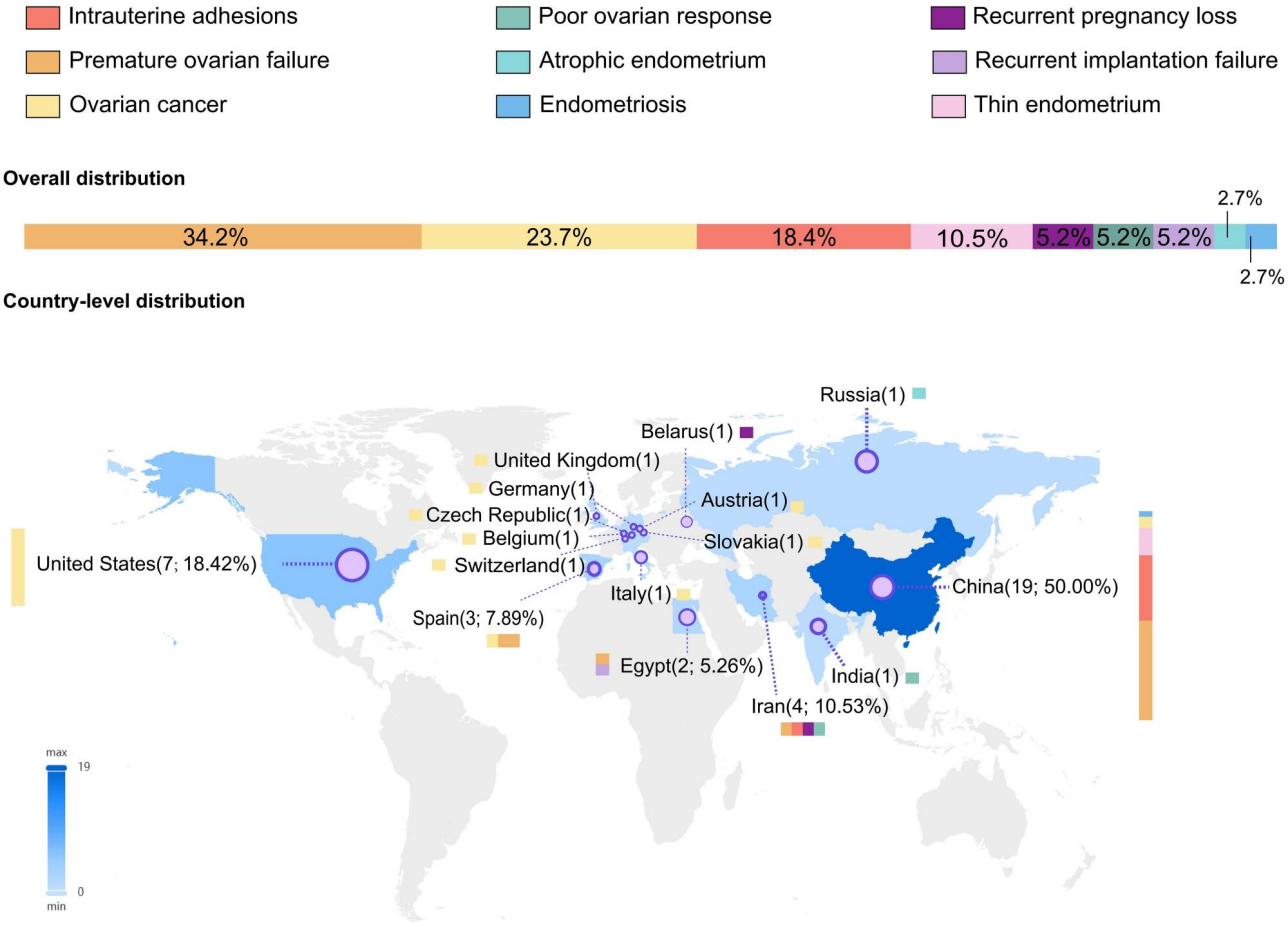
**Randomised controlled trials evaluating stem cell-based therapy in treating women reproductive diseases across countries and specialties**.

Thirty-seven (97.4%) of 38 RCTs were listed as being conducted in a single country, with China listed as conducting the most trials (19 [51.4%]), followed by the USA (7 [18.9%]). The two countries differ significantly in RCT specialties. Trials listed to be conducted in China were predominantly related to POF [9 (47.4%) of 19 trials] and IUA [6 (31.6%) of 19 trials], whereas trials listed to be conducted in the USA were all related to ovarian cancer [7 (100%) of the 7 trials]. RCTs were predominantly conducted in a single center [30 (81.1%) of 37) and included a median of 40 patients (IQR 20−120) in their final analysis. Furthermore, most of the multi-centered trials were still limited to only one country. Figure 2 highlights the distribution of trials across countries and specialties.

**Figure 3.**
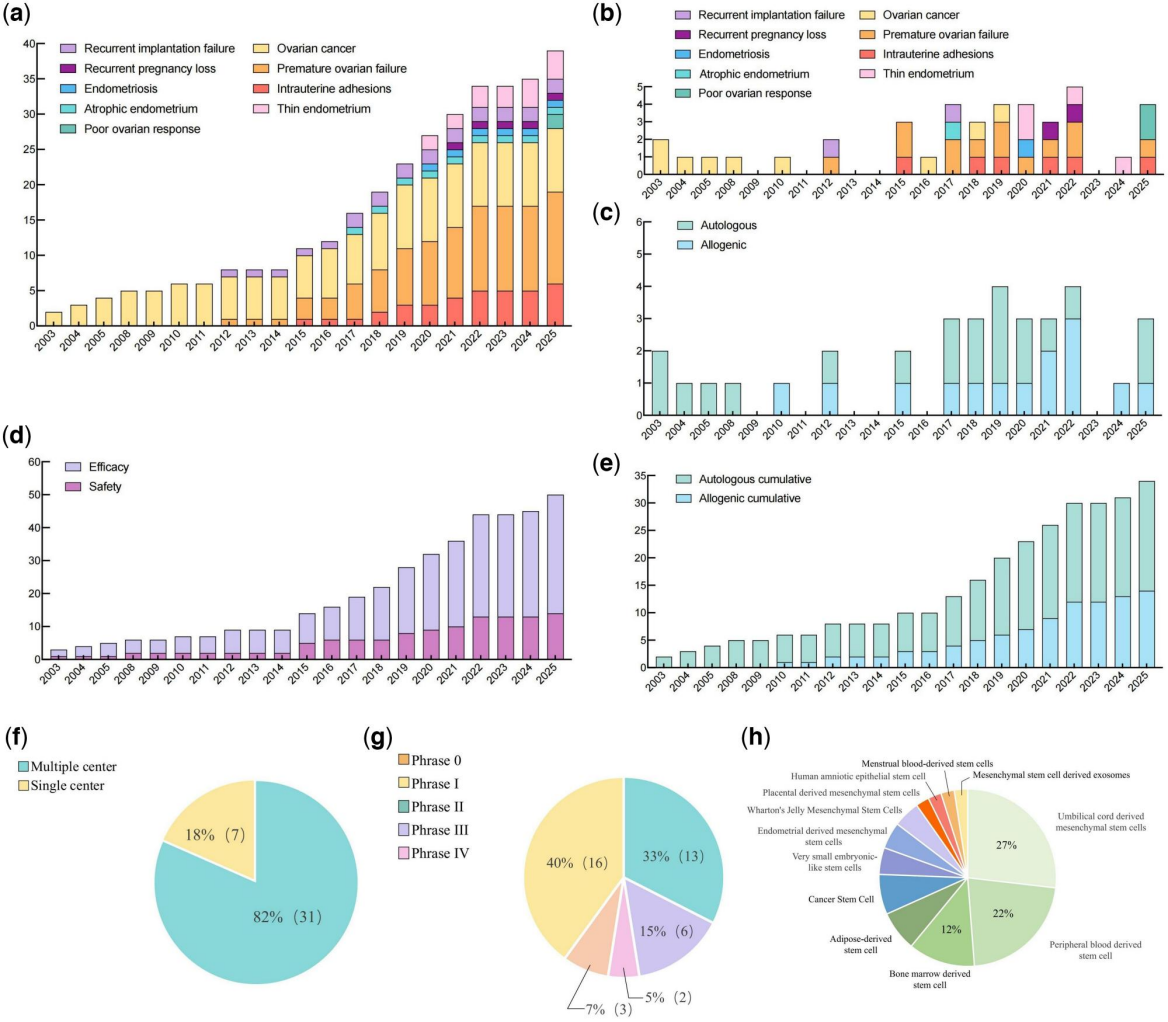
**Overview of randomised controlled trials (RCTs) evaluating stem cell-based therapy in treating women’s reproductive diseases.** (**a**) Cumulative number of initiated RCTs from 2003 to 2025, color coded for disease domains; (**b**) Number of RCTs initiated per year, color coded for disease domain; (**c**) Number of RCTs initiated per year, color coded for disease domain, color coded for cell source; (**d**) Cumulative number of initiated RCTs from 2003 to 2025, color coded for primary outcome measure; (**e**) Cumulative number of initiated RCTs from 2003 to 2025, color coded for cell source; (**f**) Pie chart showing center design of RCTs; (**g**) Pie chart showing clinical phase of RCTs; (**h**) Pie chart showing stem cell types in RCTs.

As demonstrated in Fig. 3, we examined the number of registered RCTs by year, with the earliest relevant registration being published in 2003. Between 2015 and 2022, publication numbers exhibited a gradual upward trend, signifying an emerging interest in POF. In summary, the field has exhibited momentum in worldwide registration since 2003, with new registry of RCTs every year.

Among the 38 RCTs, Phase 1 trials predominated (16 trials, 42.1%), followed by Phase 2 (13 trials, 34.2%). Only six trials (15.8%) reached Phase 3, the final stage before potential commercialization. Two Phase 4 trials (5.3%) are currently evaluating premature ovarian failure (POF) treatments. Additionally, three trials (7.9%) were designated Phase 0, an exploratory stage enabled by the FDA’s 2006 IND guidance that facilitates early human assessment of drug–target interactions and pharmacokinetics to potentially reduce later-stage attrition (Burt *et al.*, 2020). All 38 trials published since 2003 included primary endpoints related to efficacy. Safety was a co-primary endpoint in 14 trials (36.8%) and had risen from 28.6% of RCTs in 2019 to 42.9% in 2022. Umbilical cord-derived mesenchymal stem cells (UCMSC) accounted for 11 (28.9%) of all RCTs, followed by peripheral blood-derived stem cells (9, 23.7%) and bone marrow-derived stem cells (5, 13.2%). Examination of stem cell sources revealed a rise in the application of allogeneic-derived stem cells.

**Figure 4.**
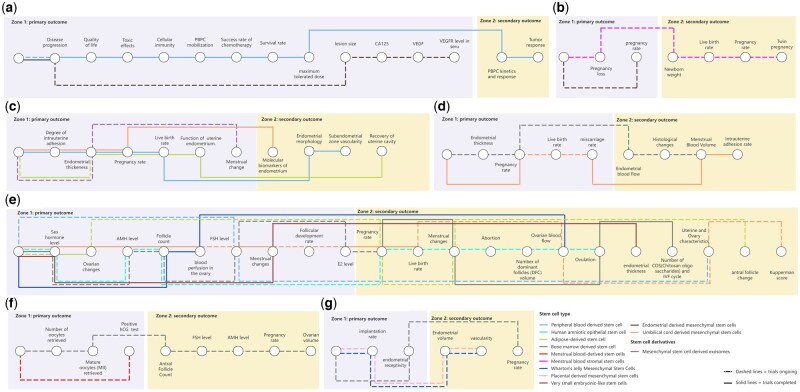
**Landscape of current stem cell-based RCTs in women’s reproductive diseases.** (**a**) Ovarian epithelial cancer; (**b**) Recurrent pregnancy loss; (**c**) Intrauterine adhesions; (**d**) Thin endometrium; (**e**) Premature ovarian failure; (**f**) Poor ovarian response; (**g**) Recurrent implantation failure. AMH, anti-Müllerian hormone; E2, estradiol.

Figure 4 summarizes the current landscape of registered RCTs investigating regenerative therapies for women’s reproductive diseases. The majority of these trials were ongoing at the time of data collection. IUA and POF represent the most frequently targeted conditions. Significant heterogeneity exists in the primary and secondary endpoints selected across these studies, with minimal overlap in core outcome measures even among trials focused on the same disease.

### Bibliometric analysis for bench-to-bedside translation

**Figure 5.**
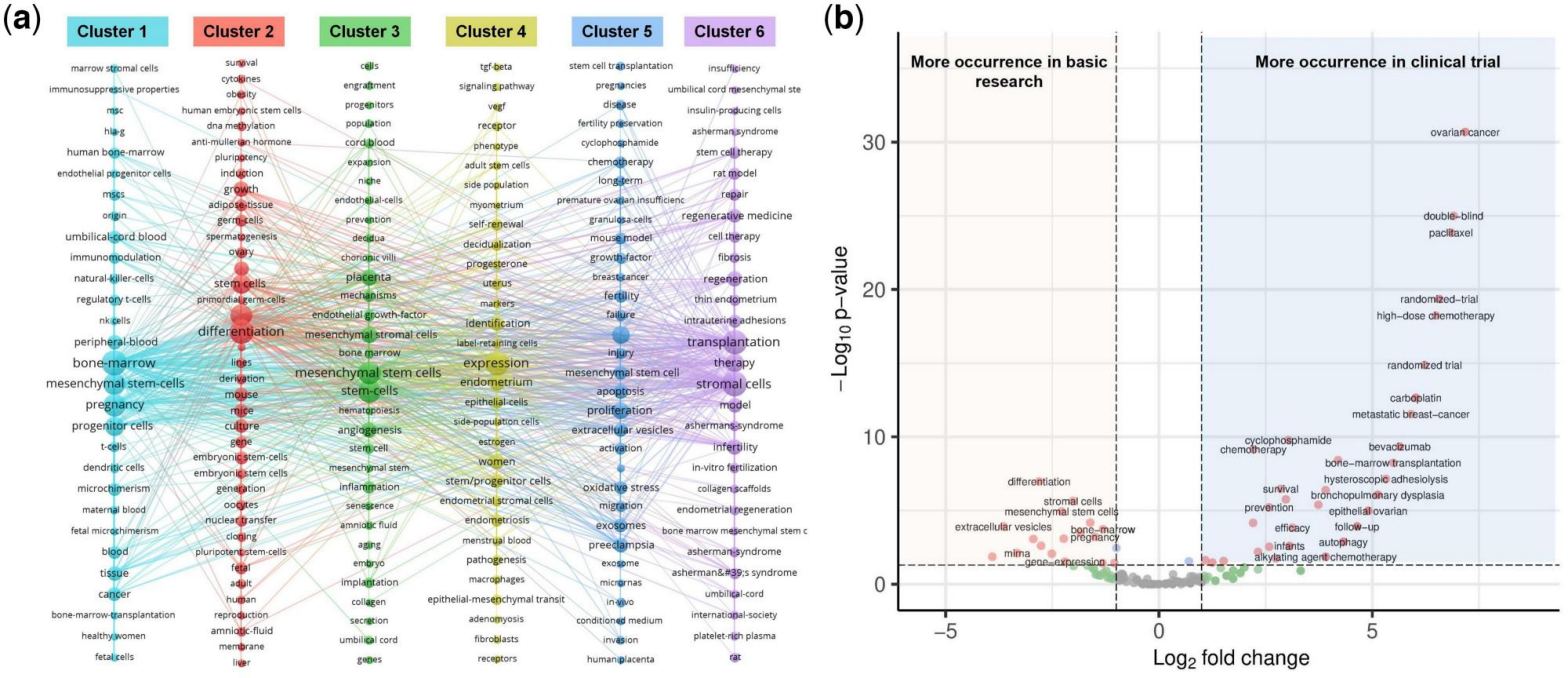
**Co-occurrence of keywords.** (**a**) Keyword co-occurrence network; (**b**) Illustration of keyword dominance.

The keywords were filtered from included basic research, and imported into VoSviewer for clustering analysis. A total of six clusters were obtained (see Fig. 5a).

Cluster 1 (Cell types and sources) outlines the foundational elements—mesenchymal stem cells (MSCs) and their origins (bone marrow, umbilical cord, adipose tissue)—emphasizing the importance of cell selection. Cluster 2 (Differentiation and growth) covers key biological processes like differentiation, pluripotency, and proliferation, essential for tissue repair. Cluster 3 (Mechanisms and molecular expression) explores the molecular underpinnings—gene expression, signaling pathways, and interactions—that drive stem cell function and therapeutic effects such as immunomodulation. Cluster 4 (Endometrial and uterine biology) provides tissue-specific context, focusing on the endometrium and uterine environment critical for successful engraftment. Cluster 5 (Transplantation and therapeutic translation) bridges research and clinical application, detailing delivery methods and regeneration strategies. Cluster 6 (Disease targets and novel treatment) highlights specific reproductive pathologies addressed by stem cell therapies and some novel combinational therapies such as ‘platelet-rich-plasma’ and ‘collagen scaffold’.

According to keyword dominance analysis of basic research and clinical trial material (Fig. 5b and Supplementary Table S3), the following terms showed statistically significant higher frequency in basic research (*P* < 0.05, fold change > 2): differentiation, pluripotency, gene expression, signaling pathway, mouse model, rat model, *in vivo*, immunomodulation, extracellular vesicle, senescence, and Wnt signaling pathway. Conversely, the following terms were significantly more prevalent in clinical trials (*P* < 0.05, fold change > 2): ovarian cancer, metastatic breast-cancer, epithelial ovarian, thin endometrium, IUA, randomized trial, double-blind, high-dose chemotherapy, follow-up, paclitaxel, carboplatin, bevacizumab, cyclophosphamide, bone-marrow transplantation, efficacy, survival, and pregnancy.

**Figure 6.**
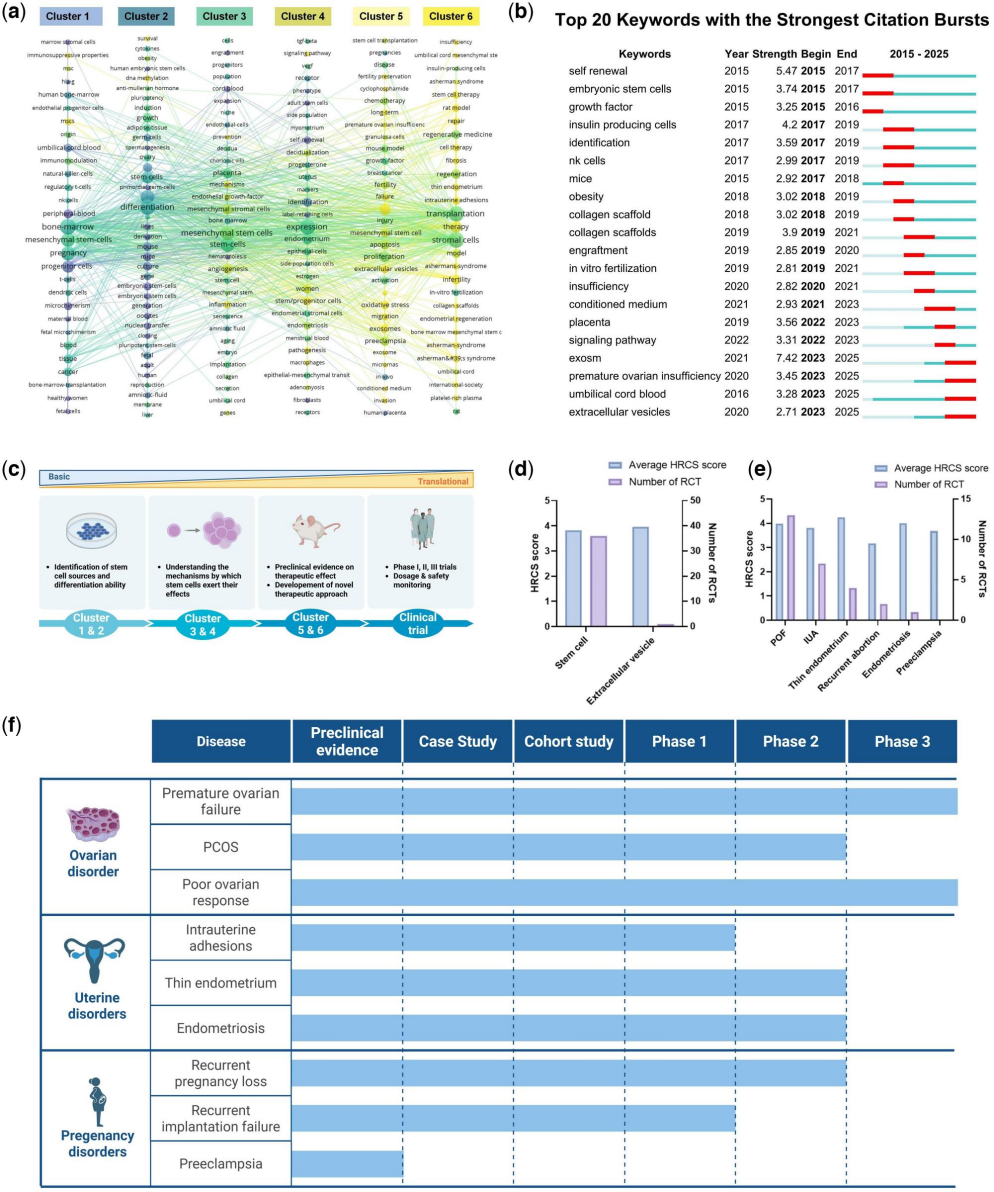
**Overview of the clinical translation of stem cell-based therapy.** (**a**) Keyword evolution over time; (**b**) Top 20 keywords with the most robust citation bursts; (**c**) Illustration of the translational trend of stem cell-based therapy for women’s reproductive diseases; (**d**) HRCS score of stem cell and extracellular vesicles; (**e**) HRCS score by disease domain; (**f**) Current stages of clinical translation by disease domain.

Temporal keyword evolution (Fig. 6a) showed that early publications (blue-coded) focused on terms related to cell sourcing (umbilical cord blood, bone marrow, adipose tissue) and core biological properties (pluripotency, self-renewal, differentiation). Later publications (yellow-coded) increasingly featured terms associated with applied research and therapeutic development. Yet, this visualization alone remains limited because it only tracks the volume of annual publications for each keyword and does not capture the abrupt surges or declines in scholarly attention that often herald a new direction.

To overcome this limitation, we employed CiteSpace’s burst-detection algorithm to extract citation bursts for all keywords, focusing especially on the top 20 that exhibited the strongest temporal accelerations. The resulting landscape, shown in Fig. 6b, uncovers the precise moments when specific terms rapidly gained traction and then quickly faded. For instance, early-phase concepts such as ‘self-renewal’ experienced pronounced bursts between 2015 and 2017 but have since plateaued, confirming that the field has largely resolved foundational questions about stem-cell identity. In contrast, keywords like ‘exosome’, ‘collagen scaffolds’, and ‘conditioned medium’ have erupted in recent years with unprecedented strength.

## Discussion

Our translational analysis reveals that stem cell-based therapies for women’s reproductive diseases—particularly POF and IUA—have gained substantial momentum in clinical trial registration. The field of stem cell-based therapy remains characterized by early-phase, single-center, and predominantly nationally confined studies. Despite robust preclinical evidence supporting therapeutic mechanisms, clinical trials exhibit marked heterogeneity in stem cell sources, dosing, outcome measures, and follow-up duration, severely limiting comparability and meta-analytic synthesis. Notably, safety, especially long-term risks such as tumorigenicity, is inadequately addressed, with nearly half of trials employing short-term monitoring.

A further examination of stem cell sources revealed a significant rise in the application of allogeneic-derived stem cells. Although autologous stem cells have the advantage of lower immunogenicity and fewer ethical concerns (Li *et al.*, 2021), recent advancements in allogeneic stem cells, particularly UCMSCs, have made them more cost-effective and commercially viable. This progress is largely due to the ability to the enablement large-scale biobanking and preservation without donor variability issues (Durand and Zubair, 2022).

While IUA and POF represent the most frequently targeted conditions, significant heterogeneity exists in the primary and secondary endpoints selected across these studies (Fig. 4). Critically, there is minimal overlap in core outcome measures, even among trials focused on the same disease. This inconsistency in outcome reporting highlights a fundamental challenge: the current lack of standardized, clinically meaningful endpoints impedes direct comparison of results and meta-analysis. Therefore, future RCTs in this field require significant refinement and harmonization of outcome measures to enable robust evaluation of therapeutic efficacy and accelerate clinical translation.

### Gaps and bridges between basic research and clinical translation

#### Gaps

Bibliometric analysis of Cluster 1 (‘Cell type and sources’) and clinical trial data both highlight a critical barrier: the absence of standardized protocols for stem cell isolation, preservation, and quality control. Key terms like ‘origin’, ‘bone-marrow’, and ‘umbilical-cord blood’ indicate that basic research emphasizes cellular heterogeneity and biological characteristics (Ong *et al.*, 2021; Cui *et al.*, 2023). However, this heterogeneity has not been adequately investigated clinically. While RCTs have examined diverse stem cell types, substantial variations in preservation methods, dosage, and quality control impede meaningful comparison of therapeutic outcomes. For instance, few RCTs report patient dosages, and those that do exhibit wide ranges—from 1 × 10^6^ to 1.5 × 10^8^ cells. Preservation practices further exacerbate this problem: some studies use freshly isolated cells (e.g. NCT05308342), while others employ cryopreserved bio-banked cells with minimally documented parameters. Moreover, despite regulatory guidelines advocating xeno-free culture to minimize immunogenicity (Cimino *et al.*, 2017), few RCTs document cultivation or quality control protocols.

Bibliometric analysis identified ‘differentiation’ as a dominant theme in Cluster 2 (‘Differentiation and growth’), highlighting another translational gap: the need for rigorous potency assays. These product-specific assays measure biological activity and therapeutic efficacy by aligning with the stem cell’s mechanism of action. Standard evaluations include *in vitro* differentiation and genetic stability assessments (Porat *et al.*, 2015). The underemphasis on such potency validation in clinical settings delays therapeutic adoption. Additionally, basic research consistently demonstrates that stem cell signaling pathways are intricately involved not only in regeneration but also in cancer development. The potential risk, though likely low, extends to rare outcomes such as ovarian granulosa cell tumors arising from treated stromal cells (Nogales *et al.*, 2018; Liu *et al.*, 2023). Crucially, while these oncogenic risks represent a major safety concern derived from basic science, they remain inadequately assessed in the clinical sphere. Most RCTs feature only short-term follow-up (18/37, 49%), leaving long-term tumorigenic observations largely elusive.

In addition, ‘international society’ mentioned in Cluster 6 reveals another major hurdle: the lack of international cooperation and guidelines for stem cell-based therapy in women’s reproductive diseases. Most RCTs are single-country studies with limited participants, restricting result generalizability. While technical standards are emerging (e.g. ISO 22859 for NCT05495711), regulatory approaches vary drastically across nations and are complicated by the sensitive nature of reproductive health (Cucinella *et al.*, 2024). The USA and EU employ flexible pathways (e.g. ‘361 products’, conditional approval) that prioritize accelerated access but may reduce incentives for robust RCTs (Rosemann *et al.*, 2016). Conversely, China and India enforce stringent regulations ensuring high local standards (Gao and Gao, 2022; Nair *et al.*, 2025). This lack of global harmonization fundamentally hinders the generation of large-scale, universally applicable evidence.

#### Bridges

Despite persistent translational challenges, significant progress across multiple domains is accelerating the clinical advancement of stem cell therapies for women’s reproductive diseases. Foundational insights into molecular mechanisms have transitioned from descriptive biology toward enabling rational therapeutic design, particularly signaling pathways like Wnt/β-catenin, PI3K/Akt, and immunomodulatory cascades highlighted in Cluster 3 (Khalil and Altayfa, 2025). Building on these mechanistic insights, synergistic combinatorial strategies foreshadowed in Cluster 6 (e.g. ‘platelet-rich plasma’, ‘collagen scaffold’) now demonstrate tangible clinical promise. For instance, RCTs such as ChiCTR2000029267 integrate stem cells with adjuvants like PRP (Éliás *et al.*, 2024), which is rich in regenerative factors (VEGF, PDGF, IGF-1), or other specific growth factors identified through mechanistic studies. This paradigm amplifies therapeutic efficacy through multiplicative effects while potentially lowering required cell doses, thereby alleviating manufacturing burdens and safety concerns.

Additionally, as evidenced in Fig. 4, the current clinical trial landscape is firmly grounded in robust preclinical evidence. This strong foundational research provides crucial validation for therapeutic potential and de-risks early clinical translation. RCTs on POF exemplifies this pipeline: a substantial volume of preclinical studies across diverse models has consistently demonstrated the capacity of MSCs to restore ovarian function, improve folliculogenesis, and modulate the ovarian microenvironment through paracrine mechanisms and immunomodulation (Zhao *et al.*, 2020). This compelling preclinical efficacy directly underpins the initiation of numerous registered clinical trials targeting POF with similar endpoints (e.g. NCT04475744, NCT03985462). Similarly, conditions like IUA and thin endometrium benefit from extensive preclinical data showing stem cell-enhanced endometrial regeneration, angiogenesis, and fertility restoration (Hong, 2024). The translation of these preclinical findings into active clinical investigation represents a critical bridge, demonstrating the field’s capacity to systematically advance promising therapies based on rigorous biological evidence.

### Emerging future research foci

#### Acellular stem cell therapy

The prominence of ‘exosome’ research signifies a strategic shift toward acellular strategies that eliminate the need for direct transplantation (Hoang *et al.*, 2022). Compared to cellular therapies, acellular approaches offer advantages: mitigating ethical concerns, reducing immunogenicity, simplifying logistics, and lowering tumorigenicity risks (Su *et al.*, 2024). Preclinical evidence demonstrates therapeutic potential—e.g. UCMSC exosomes restored ovarian function in aging mice via miR-21-5p (Li *et al.*, 2023a). HRCS scoring confirms translational potential parallels whole cells (Fig. 6d and Supplementary Table S4). However, only one registered RCT (ChiCTR2200062678) currently assesses extracellular vesicle (EV) efficacy, underscoring the urgent need for clinical validation.

#### Biomaterial combinations

The prominence of ‘collagen scaffolds’ exemplifies the rise of advanced biomaterial strategies addressing delivery challenges through enhanced cell retention and structural support (Xin *et al.*, 2019; Huang *et al.*, 2024). Initial clinical trials are emerging: a recent RCT demonstrated non-significant trends toward improved pregnancy rates using hUCMSC-loaded scaffolds in thin endometrium (Hou *et al.*, 2025), while similar approaches in POF have restored ovarian function and achieved pregnancies (Ding *et al.*, 2018). However, clinical adoption remains nascent, requiring larger confirmatory trials to establish efficacy and long-term safety.

#### Standardized preconditioning strategies

The emergence of ‘conditioned medium’ signifies a strategic focus on optimizing therapeutic potency prior to administration through molecular priming (e.g. hypoxia preconditioning upregulating HIF-1α/PI3K-Akt). While some RCTs have integrated stem cell pretreatment (e.g. NCT00019916), standardized protocols and quantitative efficacy metrics remain lacking. Regulatory ambiguities in categorizing manipulated cells as ‘minimally manipulated’ create perverse incentives that stifle innovation (Chisholm *et al.*, 2017; Di Matteo *et al.*, 2019), necessitating international harmonization efforts.

#### Broadening of disease research

Current trials concentrate disproportionately on POF (Fig. 4), yet HRCS scoring identifies IUA, endometriosis, and thin endometrium as having comparable translational potential (Fig. 6e). This disparity between preclinical promise and clinical investigation represents a missed opportunity—expanding trials to these understudied conditions should be a priority. This disparity between the high promise indicated by preclinical metrics and the limited clinical investigation beyond POF underscores a critical gap in the field. Expanding clinical trial initiatives to encompass this broader spectrum of reproductive pathologies is essential to fully realize the therapeutic potential of advanced regenerative strategies, including EVs, biomaterial combinations, and optimized preconditioning protocols. Future research must prioritize diversification to address the wider landscape of reproductive health challenges.

### Toward a coordinated translational future

To transform promising preclinical advances into reliable, scalable, and equitable regenerative therapies for women’s reproductive health, we propose a coordinated 4-dimension CORE framework that addresses the systemic gaps identified in our analysis:

***C—Consensus on Outcomes and Protocols*:** Establish disease-specific core outcome sets (e.g. via COMET or WHO initiatives) and ISO-compliant standards for cell sourcing, potency, and release criteria.

***O—Opportunity Diversification*:** Expand trials beyond POF to high-need, high-potential conditions like endometriosis and thin endometrium, guided by HRCS-based prioritization and patient-centered unmet needs.

***R—Regulatory Harmonization*:** Foster alignment through international bodies (e.g. ICMRA, ICH) to standardize long-term safety monitoring (especially oncogenic risk) and enable multicenter RCTs with mutual recognition of data.

***E—Evidence Collaboration*:** Create global consortia to pool data, biospecimens, and protocols; support adaptive trial designs, shared controls, and long-term registries; and ensure inclusion of low- and middle-income countries to enhance equity and generalizability.

### Limitations of the analysis

Our study also had limitations that warrant discussion. (i) Although a substantial number of studies were included, the overall quality of the included literature was unclear, which might lead to some bias in the result. (ii) Only English and Chinese language-based RCTs were included, and papers in other languages failed to be included in the study, therefore the RCT landscape may present with certain bias. (iii) Many of the trials listed remained incomplete at the time of writing, so our reporting draws not only on scientific publications but also on clinical trial updates and interim results disseminated via press releases and abstracts from sponsors. It should be noted that the data from press releases and abstracts had not undergone scientific peer review and should therefore be interpreted with caution.

## Conclusion

In conclusion, while stem cell-based therapies demonstrate significant potential for treating women’s reproductive diseases, bridging the gap between preclinical promise and clinical application requires broader international collaboration, standardized protocols, and deeper mechanistic validation. Our translational analysis reveals that overcoming critical barriers, including inconsistent cell manufacturing practices, inadequate long-term safety assessment, and fragmented regulatory landscapes, will be essential to harness the full therapeutic value of these approaches. Despite some inherent limitations, this study provides actionable insights for future research: prioritizing harmonized trial designs, diversifying clinical targets beyond current foci, and accelerating the translation of emerging strategies like extracellular vesicles and biomaterial combinations. Addressing these challenges systematically will be crucial to transform regenerative potential into effective, accessible therapies for global reproductive health.

## Supplementary Material

hoag024_Supplementary_Data

## Data Availability

The datasets used and/or analyzed during the current study are available in the supplementary material.
