# Supplementary material for: Bridging the gap: meta-epidemiological analysis on the clinical translation of stem cell-based therapies in women’s reproductive diseases
Source: Hum Reprod Open. 2026 Mar 21;2026(2):hoag024. doi: 10.1093/hropen/hoag024 (PMC13044756; doi:10.1093/hropen/hoag024)
Supplement: hoag024_Supplementary_Data [file hoag024_supplementary_data.docx]

**Supplementary material**

**Supplementary File S1. Search strategy**

**Supplementary Table S1. Consistency measurement**

**Supplementary Table S2. Detailed information for included RCTs**

**Supplementary Table S3. Detailed data for keyword dominance graph**

**Supplementary Table S4. Detailed data for HRCS score**

**Supplementary File S1. Search strategy**

- 1. **Search strategy for randomized controlled trials**

Databases: ICTRP, Pubmed, WOSCC, Clinicaltrial.gov, ChiCTR, and EudraCT

Date Run: 2025/12/01 (YYYY/MM/DD)

**ICTRP**

| No. | Query | Results |
| --- | --- | --- |
| #1Female reproduction | ("female reproduct*" OR "women reproduct*" OR "woman reproduct*" OR "female fertilit*" OR "women fertilit*" OR "woman fertilit*" OR "female infertil*" OR "women infertil*" OR "woman infertil*" OR "female gynecolog*" OR "women gynecolog*" OR "woman gynecolog*" OR "female obstetric*" OR "women obstetric*" OR "woman obstetric*" OR "female menstrua*" OR "women menstrua*" OR "woman menstrua*" OR "female ovar*" OR "women ovar*" OR "woman ovar*" OR "female uter*" OR "women uter*" OR "woman uter*" OR "female cervi*" OR "women cervi*" OR "woman cervi*" OR "female endometr*" OR "women endometr*" OR "woman endometr*" OR "female fallopian*" OR "women fallopian*" OR "woman fallopian*" OR "female vagin*" OR "women vagin*" OR "woman vagin*" OR "female menopaus*" OR "women menopaus*" OR "woman menopaus*" OR "female contracept*" OR "women contracept*" OR "woman contracept*" OR "female hormon*" OR "women hormon*" OR "woman hormon*" OR "female pregnan*" OR "women pregnan*" OR "woman pregnan*" OR "female gestat*" OR "women gestat*" OR "woman gestat*" OR "female maternal*" OR "women maternal*" OR "woman maternal*" OR "female feta*" OR "women feta*" OR "woman feta*" OR "female embry*" OR "women embry*" OR "woman embry*" OR "female oocyt*" OR "women oocyt*" OR "woman oocyt*" OR "female gamet*" OR "women gamet*" OR "woman gamet*" OR "female IVF" OR "women IVF" OR "woman IVF" OR "female in vitro fertiliz*" OR "women in vitro fertiliz*" OR "woman in vitro fertiliz*" OR "female ART" OR "women ART" OR "woman ART" OR "female assisted reproduct*" OR "women assisted reproduct*" OR "woman assisted reproduct*" OR "female PCOS" OR "women PCOS" OR "woman PCOS" OR "female polycystic ovar*" OR "women polycystic ovar*" OR "woman polycystic ovar*" OR "female endometrios*" OR "women endometrios*" OR "woman endometrios*" OR "female pelvic inflammatory" OR "women pelvic inflammatory" OR "woman pelvic inflammatory" OR "female PID" OR "women PID" OR "woman PID" OR "female STI" OR "women STI" OR "woman STI" OR "female sexually transmitted" OR "women sexually transmitted" OR "woman sexually transmitted" OR "female HPV" OR "women HPV" OR "woman HPV" OR "female human papillomavirus" OR "women human papillomavirus" OR "woman human papillomavirus" OR "preeclampsia" OR "pre-eclampsia" OR "gestational diabetes" OR "GDM" OR "placenta* disorder*" OR "preterm birth" OR "premature birth" OR "ovarian cancer" OR "ovarian neoplas*" OR "endometrial cancer" OR "uterine neoplas*" OR "cervical cancer" OR "gestational trophoblastic disease" OR "hyperprolactinemia" OR "POI" OR "premature ovarian insufficiency" OR "amenorrhea" OR "hypothalamic amenorrhea" OR "uterine malformation*" OR "Mülleriananomaly*" OR "Asherman syndrome" OR "intrauterine adhesion*" OR "recurrent pregnancy loss" OR "habitual abortion" OR "obstetric fistula" OR "pelvic floor disorder*") | 15,568 |
| #2Stem cell | ((("stem cell*" OR "stem-cell*" OR "progenitor cell*" OR "precursor cell*") NOT ("red blood cell*" OR "white blood cell*" OR "immune cell*" OR "liver cell*"))OR("VSEL*" OR "iPS cell*")) | 10,911 |
| #3Combined concepts | #1 AND #2 | 139 |

**Pubmed**

| No. | Query | Results |
| --- | --- | --- |
| #1Female reproduction | ((("female reproduct*"[Title/Abstract] OR "women reproduct*"[Title/Abstract] OR "woman reproduct*"[Title/Abstract] OR "female fertilit*"[Title/Abstract] OR "women fertilit*"[Title/Abstract] OR "woman fertilit*"[Title/Abstract] OR "female infertil*"[Title/Abstract] OR "women infertil*"[Title/Abstract] OR "woman infertil*"[Title/Abstract] OR "female gynecolog*"[Title/Abstract] OR "women gynecolog*"[Title/Abstract] OR "woman gynecolog*"[Title/Abstract] OR "female obstetric*"[Title/Abstract] OR "women obstetric*"[Title/Abstract] OR "woman obstetric*"[Title/Abstract] OR "female menstrua*"[Title/Abstract] OR "women menstrua*"[Title/Abstract] OR "woman menstrua*"[Title/Abstract] OR "female ovar*"[Title/Abstract] OR "women ovar*"[Title/Abstract] OR "woman ovar*"[Title/Abstract] OR "female uter*"[Title/Abstract] OR "women uter*"[Title/Abstract] OR "woman uter*"[Title/Abstract] OR "female cervi*"[Title/Abstract] OR "women cervi*"[Title/Abstract] OR "woman cervi*"[Title/Abstract] OR "female endometr*"[Title/Abstract] OR "women endometr*"[Title/Abstract] OR "woman endometr*"[Title/Abstract] OR "female fallopian*"[Title/Abstract] OR "women fallopian*"[Title/Abstract] OR "woman fallopian*"[Title/Abstract] OR "female vagin*"[Title/Abstract] OR "women vagin*"[Title/Abstract] OR "woman vagin*"[Title/Abstract] OR "female menopaus*"[Title/Abstract] OR "women menopaus*"[Title/Abstract] OR "woman menopaus*"[Title/Abstract] OR "female contracept*"[Title/Abstract] OR "women contracept*"[Title/Abstract] OR "woman contracept*"[Title/Abstract] OR "female hormon*"[Title/Abstract] OR "women hormon*"[Title/Abstract] OR "woman hormon*"[Title/Abstract] OR "female pregnan*"[Title/Abstract] OR "women pregnan*"[Title/Abstract] OR "woman pregnan*"[Title/Abstract] OR "female gestat*"[Title/Abstract] OR "women gestat*"[Title/Abstract] OR "woman gestat*"[Title/Abstract] OR "female maternal*"[Title/Abstract] OR "women maternal*"[Title/Abstract] OR "woman maternal*"[Title/Abstract] OR "female feta*"[Title/Abstract] OR "women feta*"[Title/Abstract] OR "woman feta*"[Title/Abstract] OR "female embry*"[Title/Abstract] OR "women embry*"[Title/Abstract] OR "woman embry*"[Title/Abstract] OR "female oocyt*"[Title/Abstract] OR "women oocyt*"[Title/Abstract] OR "woman oocyt*"[Title/Abstract] OR "female gamet*"[Title/Abstract] OR "women gamet*"[Title/Abstract] OR "woman gamet*"[Title/Abstract] OR "female IVF"[Title/Abstract] OR "women IVF"[Title/Abstract] OR "woman IVF"[Title/Abstract] OR "female in vitro fertiliz*"[Title/Abstract] OR "women in vitro fertiliz*"[Title/Abstract] OR "woman in vitro fertiliz*"[Title/Abstract] OR "female ART"[Title/Abstract] OR "women ART"[Title/Abstract] OR "woman ART"[Title/Abstract] OR "female assisted reproduct*"[Title/Abstract] OR "women assisted reproduct*"[Title/Abstract] OR "woman assisted reproduct*"[Title/Abstract] OR "female PCOS"[Title/Abstract] OR "women PCOS"[Title/Abstract] OR "woman PCOS"[Title/Abstract] OR "female polycystic ovar*"[Title/Abstract] OR "women polycystic ovar*"[Title/Abstract] OR "woman polycystic ovar*"[Title/Abstract] OR "female endometrios*"[Title/Abstract] OR "women endometrios*"[Title/Abstract] OR "woman endometrios*"[Title/Abstract] OR "female pelvic inflammatory"[Title/Abstract] OR "women pelvic inflammatory"[Title/Abstract] OR "woman pelvic inflammatory"[Title/Abstract] OR  "female PID"[Title/Abstract] OR "women PID"[Title/Abstract] OR "woman PID"[Title/Abstract] OR "female STI"[Title/Abstract] OR "women STI"[Title/Abstract] OR "woman STI"[Title/Abstract] OR "female sexually transmitted"[Title/Abstract] OR "women sexually transmitted"[Title/Abstract] OR "woman sexually transmitted"[Title/Abstract] OR "female HPV"[Title/Abstract] OR "women HPV"[Title/Abstract] OR "woman HPV"[Title/Abstract] OR "female human papillomavirus"[Title/Abstract] OR "women human papillomavirus"[Title/Abstract] OR "woman human papillomavirus"[Title/Abstract] OR "preeclampsia"[Title/Abstract] OR "pre-eclampsia"[Title/Abstract] OR "gestational diabetes"[Title/Abstract] OR "GDM"[Title/Abstract] OR "placenta* disorder*"[Title/Abstract] OR "preterm birth"[Title/Abstract] OR "premature birth"[Title/Abstract] OR "ovarian cancer"[Title/Abstract] OR "ovarian neoplas*"[Title/Abstract] OR "endometrial cancer"[Title/Abstract] OR "uterine neoplas*"[Title/Abstract] OR "cervical cancer"[Title/Abstract] OR "gestational trophoblastic disease"[Title/Abstract] OR "hyperprolactinemia"[Title/Abstract] OR "POI"[Title/Abstract] OR "premature ovarian insufficiency"[Title/Abstract] OR "amenorrhea"[Title/Abstract] OR "hypothalamic amenorrhea"[Title/Abstract] OR "uterine malformation*"[Title/Abstract] OR "Müllerian anomaly*"[Title/Abstract] OR "Asherman syndrome"[Title/Abstract] OR "intrauterine adhesion*"[Title/Abstract] OR "recurrent pregnancy loss"[Title/Abstract] OR "habitual abortion"[Title/Abstract] OR "obstetric fistula"[Title/Abstract] OR "pelvic floor disorder*"[Title/Abstract])) | 335,870 |
| #2Stem cell | (("stem cell*"[Title/Abstract] OR "stem-cell*"[Title/Abstract] OR "progenitor cell*"[Title/Abstract] OR "precursor cell*"[Title/Abstract]) NOT ("red blood cell*"[Title/Abstract] OR "white blood cell*"[Title/Abstract] OR "immune cell*"[Title/Abstract] OR "liver cell*"[Title/Abstract])) OR ("VSEL*"[Title/Abstract] OR "iPS cell*"[Title/Abstract] OR "iPSC*"[Title/Abstract]) OR ("Stem Cells"[Mesh] OR "Stem Cell Transplantation"[Mesh] OR "Pluripotent Stem Cells"[Mesh] OR "Mesenchymal Stem Cells"[Mesh] OR "Hematopoietic Stem Cells"[Mesh] OR "Neural Stem Cells"[Mesh] OR "Induced Pluripotent Stem Cells"[Mesh] OR "Embryonic Stem Cells"[Mesh] OR "Cancer Stem Cells"[Mesh]) | 551,577 |
| #3Clinical trial | "Randomized Controlled Trial"[Publication Type] OR "Controlled Clinical Trial"[Publication Type] OR (randomized[tiab] OR randomised[tiab]) AND (controlled[tiab] OR control[tiab] OR placebo[tiab]) OR random*[tiab] AND (trial[tiab] OR group*[tiab]) OR "random allocation"[Mesh] OR "double-blind method"[Mesh] OR "single-blind method"[Mesh] | 1,147,179 |
| #4Combined concepts | #1 AND #2 AND #3 | 89 |

**WOSCC**

| No. | Query | Results |
| --- | --- | --- |
| #1Female reproduction | TS=("female reproduct*" OR "women reproduct*" OR "woman reproduct*" OR "female fertilit*" OR "women fertilit*" OR "woman fertilit*" OR "female infertil*" OR "women infertil*" OR "woman infertil*" OR "female gynecolog*" OR "women gynecolog*" OR "woman gynecolog*" OR "female obstetric*" OR "women obstetric*" OR "woman obstetric*" OR "female menstrua*" OR "women menstrua*" OR "woman menstrua*" OR "female ovar*" OR "women ovar*" OR "woman ovar*" OR "female uter*" OR "women uter*" OR "woman uter*" OR "female cervi*" OR "women cervi*" OR "woman cervi*" OR "female endometr*" OR "women endometr*" OR "woman endometr*" OR "female fallopian*" OR "women fallopian*" OR "woman fallopian*" OR "female vagin*" OR "women vagin*" OR "woman vagin*" OR "female menopaus*" OR "women menopaus*" OR "woman menopaus*" OR "female contracept*" OR "women contracept*" OR "woman contracept*" OR "female hormon*" OR "women hormon*" OR "woman hormon*" OR "female pregnan*" OR "women pregnan*" OR "woman pregnan*" OR "female gestat*" OR "women gestat*" OR "woman gestat*" OR "female maternal*" OR "women maternal*" OR "woman maternal*" OR "female feta*" OR "women feta*" OR "woman feta*" OR "female embry*" OR "women embry*" OR "woman embry*" OR "female oocyt*" OR "women oocyt*" OR "woman oocyt*" OR "female gamet*" OR "women gamet*" OR "woman gamet*" OR "female IVF" OR "women IVF" OR "woman IVF" OR "female in vitro fertiliz*" OR "women in vitro fertiliz*" OR "woman in vitro fertiliz*" OR "female ART" OR "women ART" OR "woman ART" OR "female assisted reproduct*" OR "women assisted reproduct*" OR "woman assisted reproduct*" OR "female PCOS" OR "women PCOS" OR "woman PCOS" OR "female polycystic ovar*" OR "women polycystic ovar*" OR "woman polycystic ovar*" OR "female endometrios*" OR "women endometrios*" OR "woman endometrios*" OR "female pelvic inflammatory" OR "women pelvic inflammatory" OR "woman pelvic inflammatory" OR "female PID" OR "women PID" OR "woman PID" OR "female STI" OR "women STI" OR "woman STI" OR "female sexually transmitted" OR "women sexually transmitted" OR "woman sexually transmitted" OR "female HPV" OR "women HPV" OR "woman HPV" OR "female human papillomavirus" OR "women human papillomavirus" OR "woman human papillomavirus" OR "preeclampsia" OR "pre-eclampsia" OR "gestational diabetes" OR "GDM" OR "placenta* disorder*" OR "preterm birth" OR "premature birth" OR "ovarian cancer" OR "ovarian neoplas*" OR "endometrial cancer" OR "uterine neoplas*" OR "cervical cancer" OR "gestational trophoblastic disease" OR "hyperprolactinemia" OR "POI" OR "premature ovarian insufficiency" OR "amenorrhea" OR "hypothalamic amenorrhea" OR "uterine malformation*" OR "Müllerian anomaly*" OR "Asherman syndrome" OR "intrauterine adhesion*" OR "recurrent pregnancy loss" OR "habitual abortion" OR "obstetric fistula" OR "pelvic floor disorder*") | 389,921 |
| #2Stem cell | TS=(("stem cell*" OR "stem-cell*" OR "progenitor cell*" OR "precursor cell*" OR "VSEL*" OR "iPS cell*" OR "iPSC*") NOT ("red blood cell*" OR "white blood cell*" OR "immune cell*" OR "liver cell*")) | 634,451 |
| #3Clinical trial | TS=("randomized controlled trial" OR "randomised controlled trial" OR (randomized OR randomised) AND (controlled OR control OR placebo) OR random* AND (trial OR group*) OR "random allocat*" OR "double blind*" OR "single blind*") OR DT="Randomized Controlled Trial" | 1,472,598 |
| #4Combined concepts | #1 AND #2 AND #3 | 210 |

**Clinicaltrial.gov**

| No. | Query | Results |
| --- | --- | --- |
| #1Female reproduction | "female reproduct*" OR "women reproduct*" OR "woman reproduct*" OR "female fertilit*" OR "women fertilit*" OR "woman fertilit*" OR "female infertil*" OR "women infertil*" OR "woman infertil*" OR "female gynecolog*" OR "women gynecolog*" OR "woman gynecolog*" OR "female obstetric*" OR "women obstetric*" OR "woman obstetric*" OR "female menstrua*" OR "women menstrua*" OR "woman menstrua*" OR "female ovar*" OR "women ovar*" OR "woman ovar*" OR "female uter*" OR "women uter*" OR "woman uter*" OR "female cervi*" OR "women cervi*" OR "woman cervi*" OR "female endometr*" OR "women endometr*" OR "woman endometr*" OR "female fallopian*" OR "women fallopian*" OR "woman fallopian*" OR "female vagin*" OR "women vagin*" OR "woman vagin*" OR "female menopaus*" OR "women menopaus*" OR "woman menopaus*" OR "female contracept*" OR "women contracept*" OR "woman contracept*" OR "female hormon*" OR "women hormon*" OR "woman hormon*" OR "female pregnan*" OR "women pregnan*" OR "woman pregnan*" OR "female gestat*" OR "women gestat*" OR "woman gestat*" OR "female maternal*" OR "women maternal*" OR "woman maternal*" OR "female feta*" OR "women feta*" OR "woman feta*" OR "female embry*" OR "women embry*" OR "woman embry*" OR "female oocyt*" OR "women oocyt*" OR "woman oocyt*" OR "female gamet*" OR "women gamet*" OR "woman gamet*" OR "female IVF" OR "women IVF" OR "woman IVF" OR "female in vitro fertiliz*" OR "women in vitro fertiliz*" OR "woman in vitro fertiliz*" OR "female ART" OR "women ART" OR "woman ART" OR "female assisted reproduct*" OR "women assisted reproduct*" OR "woman assisted reproduct*" OR "female PCOS" OR "women PCOS" OR "woman PCOS" OR "female polycystic ovar*" OR "women polycystic ovar*" OR "woman polycystic ovar*" OR "female endometrios*" OR "women endometrios*" OR "woman endometrios*" OR "female pelvic inflammatory" OR "women pelvic inflammatory" OR "woman pelvic inflammatory" OR "female PID" OR "women PID" OR "woman PID" OR "female STI" OR "women STI" OR "woman STI" OR "female sexually transmitted" OR "women sexually transmitted" OR "woman sexually transmitted" OR "female HPV" OR "women HPV" OR "woman HPV" OR "female human papillomavirus" OR "women human papillomavirus" OR "woman human papillomavirus" OR "preeclampsia" OR "pre-eclampsia" OR "gestational diabetes" OR "GDM" OR "placenta* disorder*" OR "preterm birth" OR "premature birth" OR "ovarian cancer" OR "ovarian neoplas*" OR "endometrial cancer" OR "uterine neoplas*" OR "cervical cancer" OR "gestational trophoblastic disease" OR "hyperprolactinemia" OR "POI" OR "premature ovarian insufficiency" OR "amenorrhea" OR "hypothalamic amenorrhea" OR "uterine malformation*" OR "Müllerian anomaly*" OR "Asherman syndrome" OR "intrauterine adhesion*" OR "recurrent pregnancy loss" OR "habitual abortion" OR "obstetric fistula" OR "pelvic floor disorder*" | 19,988 |
| #2Stem cell | ("stem cell*" OR "stem-cell*" OR "progenitor cell*" OR "precursor cell*" OR "VSEL*" OR "iPS cell*" OR "iPSC*") NOT ("red blood cell*" OR "white blood cell*" OR "immune cell*" OR "liver cell*") | 10,567 |
| #3Combined concepts | #1 AND #2 | 302 |

**Chinese Clinical Trial Registry (ChiCTR)**

| No. | Query | Results |
| --- | --- | --- |
|  | Due to the platform's inability to process complex Boolean operators, Weconducted searches respectively for the following key words and merged results manually: |  |
| #1 | female reproduct | 1 |
| #2 | women health | 0 |
| #3 | gynecolog | 653 |
| #4 | obstetric | 53 |
| #5 | uterus | 31 |
| #6 | ovar | 1097 |
| #7 | endometr | 502 |
| #8 | cervi | 1461 |
| #9 | menstrua | 83 |
| #10 | menopaus | 372 |
| #11 | fertiliz | 123 |
| #12 | pregnan | 1075 |
| #13 | placenta | 112 |
| #14 | embry | 298 |
| #15 | endometrios | 136 |
| #16 | preeclampsia | 94 |
| #17 | gestational diabet | 73 |
|  | All records were exported to Excel, duplicates removed based on trial registration numbers. |  |

**EudraCT**

| No. | Query | Results |
| --- | --- | --- |
| #1Female reproduction | "female reproduct*" OR "women reproduct*" OR "woman reproduct*" OR "female fertilit*" OR "women fertilit*" OR "woman fertilit*" OR "female infertil*" OR "women infertil*" OR "woman infertil*" OR "female gynecolog*" OR "women gynecolog*" OR "woman gynecolog*" OR "female obstetric*" OR "women obstetric*" OR "woman obstetric*" OR "female menstrua*" OR "women menstrua*" OR "woman menstrua*" OR "female ovar*" OR "women ovar*" OR "woman ovar*" OR "female uter*" OR "women uter*" OR "woman uter*" OR "female cervi*" OR "women cervi*" OR "woman cervi*" OR "female endometr*" OR "women endometr*" OR "woman endometr*" OR "female fallopian*" OR "women fallopian*" OR "woman fallopian*" OR "female vagin*" OR "women vagin*" OR "woman vagin*" OR "female menopaus*" OR "women menopaus*" OR "woman menopaus*" OR "female contracept*" OR "women contracept*" OR "woman contracept*" OR "female hormon*" OR "women hormon*" OR "woman hormon*" OR "female pregnan*" OR "women pregnan*" OR "woman pregnan*" OR "female gestat*" OR "women gestat*" OR "woman gestat*" OR "female maternal*" OR "women maternal*" OR "woman maternal*" OR "female feta*" OR "women feta*" OR "woman feta*" OR "female embry*" OR "women embry*" OR "woman embry*" OR "female oocyt*" OR "women oocyt*" OR "woman oocyt*" OR "female gamet*" OR "women gamet*" OR "woman gamet*" OR "female IVF" OR "women IVF" OR "woman IVF" OR "female in vitro fertiliz*" OR "women in vitro fertiliz*" OR "woman in vitro fertiliz*" OR "female ART" OR "women ART" OR "woman ART" OR "female assisted reproduct*" OR "women assisted reproduct*" OR "woman assisted reproduct*" OR "female PCOS" OR "women PCOS" OR "woman PCOS" OR "female polycystic ovar*" OR "women polycystic ovar*" OR "woman polycystic ovar*" OR "female endometrios*" OR "women endometrios*" OR "woman endometrios*" OR "female pelvic inflammatory" OR "women pelvic inflammatory" OR "woman pelvic inflammatory" OR "female PID" OR "women PID" OR "woman PID" OR "female STI" OR "women STI" OR "woman STI" OR "female sexually transmitted" OR "women sexually transmitted" OR "woman sexually transmitted" OR "female HPV" OR "women HPV" OR "woman HPV" OR "female human papillomavirus" OR "women human papillomavirus" OR "woman human papillomavirus" OR "preeclampsia" OR "pre-eclampsia" OR "gestational diabetes" OR "GDM" OR "placenta* disorder*" OR "preterm birth" OR "premature birth" OR "ovarian cancer" OR "ovarian neoplas*" OR "endometrial cancer" OR "uterine neoplas*" OR "cervical cancer" OR "gestational trophoblastic disease" OR "hyperprolactinemia" OR "POI" OR "premature ovarian insufficiency" OR "amenorrhea" OR "hypothalamic amenorrhea" OR "uterine malformation*" OR "Müllerian anomaly*" OR "Asherman syndrome" OR "intrauterine adhesion*" OR "recurrent pregnancy loss" OR "habitual abortion" OR "obstetric fistula" OR "pelvic floor disorder*" | 2,189 |
| #2Stem cell | ("stem cell*" OR "stem-cell*" OR "progenitor cell*" OR "precursor cell*" OR "VSEL*" OR "iPS cell*" OR "iPSC*") NOT ("red blood cell*" OR "white blood cell*" OR "immune cell*" OR "liver cell*") | 1,145 |
| #3Combined concepts | #1 AND #2 | 33 |

- 1. **Search strategy for basic research**

**Web of Science Core collection**

| No. | Query | Results |
| --- | --- | --- |
| #1 | TS=(Cell Stem OR Cells Stem OR Stem Cell OR Progenitor Cells OR Cell Progenitor OR Cells Progenitor OR Progenitor Cell OR Mother Cells OR Cell Mother OR Cells Mother OR Mother Cell OR Colony-Forming Unit OR Colony Forming Unit OR Colony-Forming Units OR Colony Forming Units OR Stem Cell Mesenchymal OR Mesenchymal Stem Cell OR Stem Cells Mesenchymal OR Bone Marrow Mesenchymal Stem Cells OR Bone Marrow Mesenchymal Stem Cell OR Bone Marrow Stromal Cells OR Bone Marrow Stromal Cell OR Bone Marrow Stromal Cells Multipotent OR Multipotent Bone Marrow Stromal Cell OR Multipotent Bone Marrow Stromal Cells OR Adipose-Derived Mesenchymal Stem Cells OR Adipose Derived Mesenchymal Stem Cells OR Adipose-Derived Mesenchymal Stromal Cells OR Adipose Derived Mesenchymal Stromal Cells OR Mesenchymal Stem Cells Adipose-Derived OR Mesenchymal Stem Cells Adipose Derived OR Adipose-Derived Mesenchymal Stem Cell OR Adipose Derived Mesenchymal Stem Cell OR Adipose Tissue-Derived Mesenchymal Stem Cell OR Adipose Tissue Derived Mesenchymal Stem Cell OR Adipose Tissue-Derived Mesenchymal Stem Cells OR Adipose Tissue Derived Mesenchymal Stem Cells OR Adipose Tissue-Derived Mesenchymal Stromal Cells OR Adipose Tissue Derived Mesenchymal Stromal Cells OR Adipose Tissue-Derived Mesenchymal Stromal Cell OR Adipose Tissue Derived Mesenchymal Stromal Cell OR Mesenchymal Stromal Cells OR Mesenchymal Stromal Cell OR Stromal Cell Mesenchymal OR Stromal Cells Mesenchymal OR Multipotent Mesenchymal Stromal Cells OR Multipotent Mesenchymal Stromal Cell OR Mesenchymal Stromal Cells Multipotent OR Mesenchymal Progenitor Cell OR Mesenchymal Progenitor Cells OR Progenitor Cell Mesenchymal OR Progenitor Cells Mesenchymal OR Wharton Jelly Cells OR Wharton's Jelly Cells OR Wharton's Jelly Cell OR wharton Jelly Cells OR Bone Marrow Stromal Stem Cells OR MSC OR BMSC OR MSCs OR BMSCs OR Menstrual stem cells OR Menstrual stem cell OR EMSC OR emacs OR placenta derived mesenchymal stem cells OR placenta derived mesenchymal stem cell OR hamscs OR umbilical cord mesenchymal stem cells OR umbilical cord mesenchymal stem cell OR UC-MSC OR adipose-derived stem cells OR ADSC OR ADSCs OR Stromal Vascular Fraction OR SVF OR somatic stem cell OR somatic stem cells OR Hematopoietic stem cells OR epidermal stem cells OR mesenchymal stem cells OR muscle stem cells OR liver stem cells OR neural stem cells OR Hematopoietic stem cell OR epidermal stem cell OR mesenchymal stem cell OR muscle stem cell OR liver stem cell OR neural stem cell orius OR ESCs OR Adult Stem Cell OR Stem Cells Adult OR Adult Somatic Stem Cells OR Somatic Adult Stem Cells OR Somatic Stem Cells OR Somatic Stem Cell OR Stem Cell Somatic OR Stem Cells Somatic OR Peripheral Stem Cell OR Peripheral Stem Cells OR Stem Cell Peripheral OR Stem Cells Peripheral OR Peripheral Blood Stem Cell OR Stem Cells Embryonic OR Cell Embryonic Stem OR Cells Embryonic Stem OR Embryonic Stem Cell OR Stem Cell Embryonic OR stem cell OR stem cell OR Peripheral Blood Stem Cells OR Adult Stem Cells OR Mesenchymal Stem Cells OR Embryonic Stem Cells OR Stem) | 1,057,348 |
| #2 | TS=(Reproductive disease OR Reproductive system diseases OR Gynecologic Diseases OR Diseases, Gynecologic OR Gynecologic Disease OR Female Genital Diseases OR Premature Ovarian Failure OR Adnexal Diseases OR Hematometra OR Vulvar Diseases OR Pelvic Inflammatory Disease OR Vaginal Disease OR Uterine Diseases OR Adnexal Disease OR Asherman Syndrome OR Pregnancy OR Obstetrics OR Reproductive Sterility OR Uterine Cervical Neoplasms OR cervical neoplasm OR reproductive rights) | 624,660 |
| #3 | #2 AND #1 and Preprint Citation Index (Exclude – Database) | 16,848 |
| #4 | #3 AND Article (Document Types) AND Retracted Publication (Exclude – Document Types) | 16,178 |

Note: TS=Topic (Searches title, abstract, author keywords, and Keywords Plus.)

**Pubmed**

| No. | Query | Results |
| --- | --- | --- |
| #1 | ("Stem Cells"[MeSH Terms] OR "Progenitor Cells"[MeSH Terms] OR "Mother Cells"[MeSH Terms] OR "Colony-Forming Units"[MeSH Terms] OR "Mesenchymal Stem Cells"[MeSH Terms] OR "Bone Marrow Cells"[MeSH Terms] AND "Mesenchymal Stem Cells"[MeSH Terms] OR "Adipose Tissue"[MeSH Terms] AND "Mesenchymal Stem Cells"[MeSH Terms] OR "Wharton Jelly"[MeSH Terms] AND "Cells"[MeSH Terms] OR "Menstrual Blood"[MeSH Terms] AND "Stem Cells"[MeSH Terms] OR "Placenta"[MeSH Terms] AND "Mesenchymal Stem Cells"[MeSH Terms] OR "Umbilical Cord"[MeSH Terms] AND "Mesenchymal Stem Cells"[MeSH Terms] OR "Hematopoietic Stem Cells"[MeSH Terms] OR "Epidermal Stem Cells"[MeSH Terms] OR "Muscle Stem Cells"[MeSH Terms] OR "Liver Stem Cells"[MeSH Terms] OR "Neural Stem Cells"[MeSH Terms] OR "Embryonic Stem Cells"[MeSH Terms] OR "Adult Stem Cells"[MeSH Terms] OR "Peripheral Blood Stem Cells"[MeSH Terms] OR "Somatic Stem Cells"[MeSH Terms]) OR (Stem Cell[tiab] OR Stem Cells[tiab] OR Progenitor Cell[tiab] OR Progenitor Cells[tiab] OR Mother Cell[tiab] OR Mother Cells[tiab] OR Colony Forming Unit[tiab] OR Colony Forming Units[tiab] OR Colony-Forming Unit[tiab] OR Colony-Forming Units[tiab] OR Stem Cell Mesenchymal[tiab] OR Mesenchymal Stem Cell[tiab] OR Stem Cells Mesenchymal[tiab] OR Bone Marrow Mesenchymal Stem Cells[tiab] OR Bone Marrow Mesenchymal Stem Cell[tiab] OR Bone Marrow Stromal Cells[tiab] OR Bone Marrow Stromal Cell[tiab] OR Bone Marrow Stromal Cells Multipotent[tiab] OR Multipotent Bone Marrow Stromal Cell[tiab] OR Multipotent Bone Marrow Stromal Cells[tiab] OR Adipose-Derived Mesenchymal Stem Cells[tiab] OR Adipose Derived Mesenchymal Stem Cells[tiab] OR Adipose-Derived Mesenchymal Stromal Cells[tiab] OR Adipose Derived Mesenchymal Stromal Cells[tiab] OR Mesenchymal Stem Cells Adipose-Derived[tiab] OR Mesenchymal Stem Cells Adipose Derived[tiab] OR Adipose-Derived Mesenchymal Stem Cell[tiab] OR Adipose Derived Mesenchymal Stem Cell[tiab] OR Adipose Tissue-Derived Mesenchymal Stem Cell[tiab] OR Adipose Tissue Derived Mesenchymal Stem Cell[tiab] OR Adipose Tissue-Derived Mesenchymal Stem Cells[tiab] OR Adipose Tissue Derived Mesenchymal Stem Cells[tiab] OR Adipose Tissue-Derived Mesenchymal Stromal Cells[tiab] OR Adipose Tissue Derived Mesenchymal Stromal Cells[tiab] OR Adipose Tissue-Derived Mesenchymal Stromal Cell[tiab] OR Adipose Tissue Derived Mesenchymal Stromal Cell[tiab] OR Mesenchymal Stromal Cells[tiab] OR Mesenchymal Stromal Cell[tiab] OR Stromal Cell Mesenchymal[tiab] OR Stromal Cells Mesenchymal[tiab] OR Multipotent Mesenchymal Stromal Cells[tiab] OR Multipotent Mesenchymal Stromal Cell[tiab] OR Mesenchymal Stromal Cells Multipotent[tiab] OR Mesenchymal Progenitor Cell[tiab] OR Mesenchymal Progenitor Cells[tiab] OR Progenitor Cell Mesenchymal[tiab] OR Progenitor Cells Mesenchymal[tiab] OR Wharton Jelly Cells[tiab] OR Wharton's Jelly Cells[tiab] OR Wharton's Jelly Cell[tiab] OR wharton Jelly Cells[tiab] OR Bone Marrow Stromal Stem Cells[tiab] OR MSC[tiab] OR BMSC[tiab] OR MSCs[tiab] OR BMSCs[tiab] OR Menstrual stem cells[tiab] OR Menstrual stem cell[tiab] OR EMSC[tiab] OR emacs[tiab] OR placenta derived mesenchymal stem cells[tiab] OR placenta derived mesenchymal stem cell[tiab] OR hamscs[tiab] OR umbilical cord mesenchymal stem cells[tiab] OR umbilical cord mesenchymal stem cell[tiab] OR UC-MSC[tiab] OR adipose-derived stem cells[tiab] OR ADSC[tiab] OR ADSCs[tiab] OR Stromal Vascular Fraction[tiab] OR SVF[tiab] OR somatic stem cell[tiab] OR somatic stem cells[tiab] OR Hematopoietic stem cells[tiab] OR epidermal stem cells[tiab] OR mesenchymal stem cells[tiab] OR muscle stem cells[tiab] OR liver stem cells[tiab] OR neural stem cells[tiab] OR Hematopoietic stem cell[tiab] OR epidermal stem cell[tiab] OR mesenchymal stem cell[tiab] OR muscle stem cell[tiab] OR liver stem cell[tiab] OR neural stem cell orius[tiab] OR ESCs[tiab] OR Adult Stem Cell[tiab] OR Stem Cells Adult[tiab] OR Adult Somatic Stem Cells[tiab] OR Somatic Adult Stem Cells[tiab] OR Somatic Stem Cells[tiab] OR Somatic Stem Cell[tiab] OR Stem Cell Somatic[tiab] OR Stem Cells Somatic[tiab] OR Peripheral Stem Cell[tiab] OR Peripheral Stem Cells[tiab] OR Stem Cell Peripheral[tiab] OR Stem Cells Peripheral[tiab] OR Peripheral Blood Stem Cell[tiab] OR Stem Cells Embryonic[tiab] OR Cell Embryonic Stem[tiab] OR Cells Embryonic Stem[tiab] OR Embryonic Stem Cell[tiab] OR Stem Cell Embryonic[tiab] OR stem cell[tiab] OR stem cell[tiab] OR Peripheral Blood Stem Cells[tiab] OR Adult Stem Cells[tiab] OR Mesenchymal Stem Cells[tiab] OR Embryonic Stem Cells[tiab] OR Stem[tiab]) | 650,215 |
| #2 | ("Reproductive System Diseases"[MeSH] OR "Gynecologic Diseases"[MeSH] OR "Premature Ovarian Failure"[MeSH] OR "Adnexal Diseases"[MeSH] OR "Vulvar Diseases"[MeSH] OR "Pelvic Inflammatory Disease"[MeSH] OR "Vaginal Diseases"[MeSH] OR "Uterine Diseases"[MeSH] OR "Asherman Syndrome"[MeSH] OR "Pregnancy"[MeSH] OR "Obstetrics"[MeSH] OR "Infertility"[MeSH] OR "Uterine Cervical Neoplasms"[MeSH] OR "Reproductive Rights"[MeSH] OR "Hematometra"[MeSH]) OR ("Reproductive disease"[tiab] OR "Reproductive system diseases"[tiab] OR "Gynecologic Diseases"[tiab] OR "Diseases, Gynecologic"[tiab] OR "Gynecologic Disease"[tiab] OR "Female Genital Diseases"[tiab] OR "Premature Ovarian Failure"[tiab] OR "Adnexal Diseases"[tiab] OR "Adnexal Disease"[tiab] OR "Hematometra"[tiab] OR "Vulvar Diseases"[tiab] OR "Pelvic Inflammatory Disease"[tiab] OR "Vaginal Disease"[tiab] OR "Uterine Diseases"[tiab] OR "Asherman Syndrome"[tiab] OR "Pregnancy"[tiab] OR "Obstetrics"[tiab] OR "Reproductive Sterility"[tiab] OR "Uterine Cervical Neoplasms"[tiab] OR "cervical neoplasm"[tiab] OR "Reproductive rights"[tiab]) | 1,593,006 |
| #3 | #2 AND #1 | 18,245 |
| #4 | #3 AND NOT ("Comment" [Publication Type] OR "Editorial" [Publication Type] OR "Letter" [Publication Type] OR "Retracted Publication" [Publication Type] OR "Review" [Publication Type]) AND Filters: English, Exclude preprints | 14,572 |

**Supplementary Table S1. Consistency measurement**

**2.1. Kappa value between ASreview and researcher**

|  | | Researcher | | Sum |
| --- | --- | --- | --- | --- |
|  |  | Exclude | Include |  |
| ASreview | Exclude | 12 | 2 | 14 |
|  | Include | 0 | 36 | 36 |
| Sum | | 12 | 38 | 50 |

Kappa value calculated by SPSS: 0.896

**Supplementary Table S2. Detailed information for included RCTs.**

| **Trial registration number** | **First posted day** | **Study status** | **Sponsor** | **Disease speciality** | **Country** | **Centers** | **Sample Size** | **Primary outcome** | **Primary result** | **Stem cell type** | **Autologous/allogenic** | **Patient dose** | **Preservation technique** | **Trial Phase** | **References** | **Additional note on commercial product** | **Single treatment /combined treatment** | **Follow-up** |
| --- | --- | --- | --- | --- | --- | --- | --- | --- | --- | --- | --- | --- | --- | --- | --- | --- | --- | --- |
| NCT06503471 | 2024/7/16 | Suspended | Sclnow Biotechnology Co., Ltd. | Thin Endometrial Infertility | China | Single center | 24 | Pregnancy rate, clinical pregnancy rate, ongoing pregnancy rate, miscarriage rate and live birth rate | NA | Umbilical cord derived mesenchymal stem cells | Allogeneic | 1 × 10^7^cells (2ml) | NA | I | NA | SCLife®-UT | Combined | Short term |
| NCT05520112 | 2022/8/29 | Unknown status | Institute of Biophysics and Cell Engineering of National Academy of Sciences of Belarus | Recurrent Pregnancy Loss | Belarus | Single center | 20 | Adverse effects associated with the therapy, percent of patients with successful pregnancy | NA | Endometrial derived mesenchymal stem cells | Autologous | NA | NA | I, II | NA | NA | Single | Short term |
| NCT05495711 | 2022/8/10 | Completed | Sclnow Biotechnology Co., Ltd. | Thin Endometrial Infertility | China | Single center | 25 | Pregnancy outcome | NA | Umbilical cord derived mesenchymal stem cells | Allogeneic | 1 × 10^7^cells (2ml) | NA | I | hUC-MSCs loaded collagen scaffold for refractory thin endometrium caused by Asherman syndrome: a double-blind randomized controlled trial | Umbilical cord mesenchymal stem cell therapy (Sclnow Biotechnology) 19#iSCLife®-UT | Combined | Short term |
| NCT05308342 | 2022/4/4 | Unknown status | Li-jun Ding | Premature Ovarian Failure (POF) | china | Single center | 66 | Follicular development rate | NA | Umbilical cord derived mesenchymal stem cells | Allogeneic | 10×106 cells, 5×106 for unilateral ovarian injection | Immediately preserved and transferred for direct injection | NA | NA | NA | Combined | Short term |
| NCT05138367 | 2021/12/1 | Completed | Li-jun Ding | Premature Ovarian Failure (POF) | china | Single center | 20 | Blood perfusion in the ovary，Antral Follicle Diameter | NA | Umbilical cord derived mesenchymal stem cells，Wharton's Jelly Mesenchymal Stem Cells | Allogeneic | 2×10^7cells, 1×10^7 /400 μL for unilateral ovarian injection |  | NA | NA | NA | Combined | Short term |
| NCT04475744 | 2020/7/17 | Completed | Fundación IVI | Premature Ovarian Failure (POF) | Spain | Multicentered | 42 | Antral follicular count | NA | Bone marrow derived stem cell | Autologous | NA | NA | III | NA | NA | Combined | Short term |
| NCT04233892 | 2020/1/18 | Unknown status | Yali Hu | Endometriosis/ thin endometrium | China | Single center | 345 | Endometrial thickness，Ongoing pregnancy rate Adverse event | NA | Bone marrow derived stem cell | Allogeneic | NA | NA | I, II | NA | NA | Combined | Long term |
| NCT03985462 | 2019/6/13 | Withdrawn | Fuda Cancer Hospital, Guangzhou | Premature Ovarian Failure (POF) | China | Single center | 0 | Concentration of Blood female hormones level | NA | Very small embryonic-like stem cells | Autologous | 100,000/200,000 / 300,000 VSEL cells | NA | I, II | NA | NA | single | Short term |
| NCT03949283 | 2019/5/14 | Active, not recruiting | Cordgenics, LLC | Recurrent platinum-resistant ovarian cancer | USA | Multicentered | 150 | Objective response rate | Improved PFS and OS | Cancer Stem Cell | Autologous | NA | NA | III | Clinical relevance of cancer stem cell chemotherapeutic assay for recurrent ovarian cancer，Cancer Stem Cell Assay for the Treatment of Platinum-Resistant Recurrent Ovarian Cancer，ChemoID-guided therapy improves objective response rate in recurrent platinum-resistant ovarian cancer randomized clinical trial | NA | NA | Long term |
| NCT03816852 | 2019/1/25 | Suspended | Sclnow Biotechnology Co., Ltd. | Premature Ovarian Failure (POF) | China | Single center | 12 | Safety and efficacy,Menstrual changes | NA | Umbilical cord derived mesenchymal stem cells | Allogeneic | 9*10^7 cells,6*10^7 cells,3*10^7 cells | NA | II | NA | 19#iSCLife®-POI | single | Short term |
| NCT03632798 | 2018/8/15 | Withdrawn | Cordgenics, LLC | Recurrent epithelial ovarian cancer (EOC) | USA | Multicentered | 0 | Progression free survival (PFS) | NA | Cancer Stem Cell | Autologous | NA | NA | III | NA | ChemoID assay | Combined | Long term |
| NCT03535480 | 2018/5/24 | Unknown status | Instituto de Investigacion Sanitaria La Fe | Premature Ovarian Failure (POF) | Spain | Single center | 20 | Antral follicle count (AFC) | NA | Bone marrow derived stem cell | Autologous | NA | NA | IV | NA | NA | Combined | Long term |
| NCT03166189 | 2017/5/25 | Completed | D.O. Ott Research Institute of Obstetrics, Gynecology, and Reproductology | Atrophic Endometrium in Patients With Repeated IVF Failures | Russia | Single center | 46 | Assessment of endometrial receptivity | NA | Bone marrow derived stem cell | Autologous | 5 millions | NA | II | NA | NA | Combined | Short term |
| NCT03033277 | 2017/1/10 | Unknown status | Chinese Academy of Sciences | Premature Ovarian Failure (POF) | China | Single center | 320 | Number of mature follicle | NA | Umbilical cord derived mesenchymal stem cells | Allogeneic | NA | NA | I, II | NA | NA | single | Short term |
| NCT02783937 | 2017/1/26 | Unknown status | South Valley University | Premature Ovarian Failure (POF) | Egypt | Single center | 10 | Return of menses | NA | Very small embryonic-like stem cells | Autologous | NA | NA | IV | NA | NA | single | Short term |
| NCT02713386 | 2016/3/18 | Completed | NRG Oncology | III-IV Epithelial Ovarian, Fallopian Tube, Primary Peritoneal Cancer | USA | Multicentered | 147 | Dose-limiting Toxicities (Phase I),Progression-free Survival (PFS) (Phase II) | Ruxolitinib 15 mg PO twice a day was well tolerated with acceptable toxicity in combination with paclitaxel/carboplatin chemotherapy | Cancer Stem Cell | NA | NA | NA | I, II | Phase I and Randomized Phase II Study of Ruxolitinib With Frontline Neoadjuvant Therapy in Advanced Ovarian Cancer: An NRG Oncology Group Study | NA | Combined | Long term |
| NCT02644447 | 2015/12/31 | Completed | Chinese Academy of Sciences | Premature Ovarian Failure (POF) | China | Single center | 23 | Safety and Tolerability assessed by Adverse Events | NA | Umbilical cord derived mesenchymal stem cells | Allogeneic | bilateral ovaries injection of 10 million allogeneic HUC-MSCs | NA | I, II | NA | NA | Combined | Short term |
| NCT02603744 | 2015/11/13 | Unknown status | Royan Institute | Premature Ovarian Failure (POF) | Iran | Single center | 9 | Eficacy and safety,ovary mass,ovary abcess | The intra-ovarian embedding of ADSCs is safe and feasible and is associated with an inconsistent decline in serum FSH | Adipose-derived stem cell | Autologous | 5 million MSC,10 million MSC,15 million MSC | NA | I, II | Evaluation of safety, feasibility and efficacy of intra-ovarian transplantation of autologous adipose derived mesenchymal stromal cells in idiopathic premature ovarian failure patients: non-randomized clinical trial, phase I, first in human | NA | single | Short term |
| NCT01742533 | 2012/12/5 | Unknown status | Shenzhen Beike Bio-Technology Co., Ltd. | Premature Ovarian Failure (POF) | China | Single center | 40 | Serum Level of follicle-stimulating hormone | NA | Umbilical cord derived mesenchymal stem cells ,Peripheral blood derived stem cell | Allogeneic | NA | NA | I, II | NA | NA | Combined | Short term |
| NCT01649752 | 2012/7/25 | Unknown status | Kasr El Aini Hospital | Recurrent implantation failure | Egypt | Single center | 60 | Implantation rate | NA | Placental derived mesenchymal stem cells (MSC),Wharton's Jelly Mesenchymal Stem Cells | Autologous | NA | NA | I | NA | NA | single | Short term |
| NCT00652691 | 2008/4/4 | Completed | Mayo Clinic | Ovarian cancer | USA | Single center | 48 | Maximum tolerated dose of topotecan hydrochloride,toxicity according to NCI criteria | Topotecan can be safely dose escalated to 4.5 mg/m2 per day in combination with cyclophosphamide, carboplatin and AHSCT | Peripheral blood derived stem cell | Autologous | NA | NA | I | Phase I Trial of Autologous Hematopoietic Stem Cell Transplantation with Escalating Doses of Topotecan Combined with Cyclophosphamide and Carboplatin in Patients with Relapsed or Persistent Ovarian or Primary Peritoneal Carcinoma | NA | Combined | Long term |
| NCT00117442 | 2005/7/7 | Completed | Amgen | Breast cancer,ovarian cancer | USA | Single center | 61 | PBPC mobilization profiles and success rate of achieving planned chemotherapy administration on time | CD41a can be used as a surrogate marker for megakaryocytic mobilization | Peripheral blood derived stem cell | Autologous | NA | NA | II | Pegfilgrastim for peripheral CD34 mobilization in patients with solid tumours+,Pegfilgrastim successfully mobilizes megakaryocyte progenitors into the peripheral blood in subjects with solid tumours | NA | Combined | N/A |
| NCT00019916 | 2003/1/27 | Completed | National Cancer Institute (NCI) | Stage IV, Recurrent, or Progressive Breast or Ovarian Cancer | USA | Multicentered | 34 | Effectiveness, Cellular immunity | NA | Peripheral blood derived stem cell | Autologous | NA | NA | NA | NA | NA | Single | Long term |
| NCT00004921 | 2003/1/27 | Completed | EBMT Solid Tumors Working Party | Stage III or Stage IV Ovarian Epithelial Cancer That Has Been Removed During Surgery | Austria, Belgium, Czech Republic, Germany, Italy, Slovakia, Spain, Switzerland, UK | Multicentered | 208 | Survival, toxicity, and quality of life | Seventy-six percent of patients received all five cycles in the HD arm and the main toxicities were neuro-/ototoxicity, gastrointestinal toxicity, and infection and one death from hemorrhagic shock. After a median follow-up of 38 months, the progression-free survival was 20.5 months in the standard arm and 29.6 months in the HD arm. | Peripheral blood derived stem cell | Autologous | NA | NA | III | Mobus V, Wandt H, Frickhofen N, Bengala C, Champion K, Kimmig R, Ostermann H, Hinke A, Ledermann JA; AGO-Ovar/AIO; EBMT. Phase III trial of high-dose sequential chemotherapy with peripheral blood stem cell support compared with standard dose chemotherapy for first-line treatment of advanced ovarian cancer: intergroup trial of the AGO-Ovar/AIO and EBMT. J Clin Oncol. 2007 Sep 20;25(27):4187-93. doi: 10.1200/JCO.2006.09.7527. Epub 2007 Aug 13. | NA | Combined | Long term |
| NCT00002819 | 2004/5/26 | Terminated | Gynecologic Oncology Group | Ovarian epithelial cancer | USA | Multicentered | 275 | Effectiveness, Quality of life, disease progression | NA | Peripheral blood derived stem cell | Autologous | NA | NA | III | NA | NA | Combined | Long term |
| IRCT20210513051280N2 | 2021/5/31 | Pending | Iranian academic center for education culture and research | recurrent pregnancy loss | Iran | Single center | 40 | Safety and effectiveness, Pregnancy loss | NA | Peripheral blood derived stem cell | Allogeneic | 0.5 ml containing 1 million cells. | NA | I, II | NA | NA | Single | Short term |
| IRCT20160422027520N26 | 2025/2/3 | Completed | Tabriz University of Medical Sciences | Intrauterine adhesions | Iran | Single center | 12 | Efficacy, Intrauterine adhesion rate | NA | Umbilical cord derived mesenchymal stem cells | NA | 1 x 10^7 single-dose | NA | NA | NA | NA | Single | N/A |
| CTRI/2025/03/083537 | 2025/03/26 | Not Yet Recruiting | AIIMS New Delhi | Poor ovarian response (POR) | India | Single center | 39 | Efficacy,Number of oocytes and mature oocytes (MII) retrieved | NA | Bone marrow derived stem cell | Autologous | 2ml | NA | NA | NA | NA | Combined | Short term |
| ChiCTR-TRC-10000973 | 2010/8/1 | Completed | third military medical university | Recurrent ovarian cancer | China | Single center | 20 | Efficacy,lesion size, CA125, VEGF, VEGFR level in seru | NA | Endometrial derived mesenchymal stem cells | Allogeneic | NA | NA | NA | NA | NA | Combined | N/A |
| ChiCTR-ICC-15006046 | 2015/3/6 | Completed | Chinese National 2014 | Intrauterine adhesions | China | Single center | 20 | Efficacy and safety ,thickness, function of the uterine endometrium. | NA | Adipose-derived stem cell | NA | NA | NA | NA | NA | NA | Single | N/A |
| ChiCTR2500097156 | 2025/2/13 | Active | Women's Hospital School Of Medicine Zhejang University | Premature Ovarian Failure (POF) | China | Single center | 36 | Safety and efficacy and serum AMH and basic sex hormone levels and antral follicle count (AFC) | NA | Human amniotic epithelial stem cell | Allogeneic | NA | NA | I | NA | NA | Combined | N/A |
| ChiCTR2200062678 | 2022/8/15 | Active | Clinical Research 121 Project of Beijing Hospital | Intrauterine adhesions | China | Single center | 220 | Safety and efficacy,normal menstrual period and volume, thickeness | NA | Mesenchymal stem cell derived exosomes | NA | NA | NA | I | NA | NA | Single | Long term |
| ChiCTR2200056751 | 2022/2/13 | Completed | National key research and development plan in 2018 | Premature Ovarian Failure (POF) | China | Single center | 72 | Safety and efficacy, FSH、E2、AMH | NA | Umbilical cord derived mesenchymal stem cells | Allogeneic | NA | NA | I | NA | NA | Combined | N/A |
| ChiCTR2100052229 | 2021/10/23 | Completed | The Second Affiliated Hospital of PLA Air Force Military Medical University | Intrauterine adhesions | China | Single center | 120 | Ongoing pregnancy rate | NA | Adipose-derived stem cell | Autologous | NA | NA | 0 | NA | NA | Single | N/A |
| ChiCTR2000029267 | 2020/1/19 | Completed | Jinan Central Hospital Affiliated to Shandong University | Thin endometrium and Asherman’s syndrome | China | Single center | 500 | Adhesion score, pregnancy rate | NA | Peripheral blood derived stem cell | Autologous | NA | NA | 0 | NA | NA | Combined | N/A |
| ChiCTR1900027149 | 2019/11/2 | Completed | Shengjing Hospital affiliated to China Medical University | Intrauterine adhesions | China | Single center | 70 | Feasibility and safety, Live birth rate | NA | Peripheral blood derived stem cell | Autologous | NA | NA | 0 | NA | NA | Combined | N/A |
| ChiCTR1800018240 | 2018/9/6 | Completed | Zhejiang Medical and Health Technology Project | Intrauterine adhesions | China | Single center | 30 | Efficacy, Degree of intrauterine adhesion, endometrial thickness, pregnancy | NA | Umbilical cord derived mesenchymal stem cells | Allogeneic | NA | NA | NA | NA | NA | Combined | Short term |
| IRCT20250609066140N1 | 2025/7/13 | Pending | Alborz University of Medical Sciences | Poor ovarian response (POR) | Iran | Single center | 105 | Positive serum B-Human Chorionic Gonadotropin (hCG) test | NA | Menstrual blood-derived stem cells (MenSCs) | Autologous | NA | NA | II,III | NA | NA | Single | Short term |

**Supplementary Table S3. Detailed data for keyword dominance graph**

| **keyword** | **log 2 fold change** | **p-value** |
| --- | --- | --- |
| 1st-line treatment | 3.321928095 | 0.120869702 |
| activation | 0.428843299 | 0.635744122 |
| acute myeloid-leukemia | 3.321928095 | 0.120869702 |
| adenomyosis | 0.152003093 | 1 |
| adipose-derived stem cells | 0.736965594 | 0.873949692 |
| adjuvant treatment | 3.321928095 | 0.120869702 |
| agents | 3.321928095 | 0.120869702 |
| akt signaling pathway | 3.321928095 | 0.120869702 |
| alkylating agent chemotherapy | 3.906890596 | 0.013747874 |
| amenorrhea | 3.321928095 | 0.120869702 |
| amnion | 0.514573173 | 1 |
| amnion graft | 3.321928095 | 0.120869702 |
| anal incontinence | 3.321928095 | 0.120869702 |
| anal sphincter | 3.321928095 | 0.120869702 |
| angiogenesis | -0.847996907 | 0.213524354 |
| antenatal magnesium-sulfate | 3.321928095 | 0.120869702 |
| anthracycline-resistant | 3.321928095 | 0.120869702 |
| apoptosis | -1.552541023 | 0.083580974 |
| asherman syndrome | 1 | 0.400021035 |
| asherman's syndrome | -0.321928095 | 0.866477063 |
| ashermans-syndrome | 0.807354922 | 0.296201254 |
| asherman-syndrome | 1.251538767 | 0.034355084 |
| association | 1 | 0.73457052 |
| autologous bone-marrow | 3.321928095 | 0.120869702 |
| autologous transplantation | 3.321928095 | 0.120869702 |
| autophagy | 4.321928095 | 0.001342727 |
| balloon | 3.321928095 | 0.120869702 |
| bevacizumab | 5.64385619 | 4.63E-10 |
| birth-weight infants | 4.321928095 | 0.001342727 |
| blood | -1.137503524 | 0.256295081 |
| blood progenitor cells | 3.906890596 | 0.013747874 |
| bmscs | 1.736965594 | 0.422742778 |
| bone marrow mesenchymal stem cells | 0.321928095 | 1 |
| bone-marrow | -1.310340121 | 0.000188628 |
| bone-marrow support | 4.321928095 | 0.001342727 |
| bone-marrow transplantation | 5.491853096 | 5.79E-09 |
| bone-marrow-transplantation | 1.447458977 | 0.08048679 |
| bone-mineral density | 3.321928095 | 0.120869702 |
| brain | 0.321928095 | 1 |
| brain-injury | 3.906890596 | 0.013747874 |
| breast | 1.906890596 | 0.175032934 |
| breast cancer | 2.321928095 | 0.006435091 |
| breast-cancer | 1.514573173 | 0.026873744 |
| bronchopulmonary dysplasia | 5.129283017 | 8.75E-07 |
| cancer | 0.134301092 | 0.948487844 |
| cancer patients | 3.321928095 | 0.120869702 |
| cancer stem cells | 3.058893689 | 0.002576341 |
| cancer stem-cells | 3.906890596 | 0.013747874 |
| carboplatin | 6.022367813 | 2.24E-13 |
| carcinoma | 6.022367813 | 2.24E-13 |
| cd44 | 3.321928095 | 0.120869702 |
| cell support | 3.906890596 | 0.013747874 |
| cell therapy | -1.201633861 | 0.366052516 |
| cells | 1.058893689 | 0.274649481 |
| cervical cancer | 3.321928095 | 0.120869702 |
| cervical-cancer | 3.906890596 | 0.013747874 |
| chemoresistance | 3.906890596 | 0.013747874 |
| chemotherapy | 2.215012891 | 7.04E-10 |
| chemotherapy regimens | 3.321928095 | 0.120869702 |
| children | 1 | 0.73457052 |
| chronic lung-disease | 3.906890596 | 0.013747874 |
| chronic lymphocytic-leukemia | 3.321928095 | 0.120869702 |
| cisplatin | 6.022367813 | 2.24E-13 |
| clinical trial | 1.736965594 | 0.118250884 |
| clinical-trials | 2.736965594 | 0.017626866 |
| clomiphene citrate | 3.906890596 | 0.013747874 |
| clonal hematopoiesis | 3.321928095 | 0.120869702 |
| collagen scaffolds | 0.621488377 | 0.662135179 |
| colony-stimulating factor | 4.196397213 | 3.84E-09 |
| colony-stimulating factors | 3.321928095 | 0.120869702 |
| colorectal cancer | 3.321928095 | 0.120869702 |
| combination | 3.906890596 | 0.013747874 |
| combination chemotherapy | 3.321928095 | 0.120869702 |
| complete response | 3.321928095 | 0.120869702 |
| complications | 1.906890596 | 0.175032934 |
| conditioned media (cm) | 3.321928095 | 0.120869702 |
| consolidation | 3.321928095 | 0.120869702 |
| consolidation therapy | 3.321928095 | 0.120869702 |
| controlled-trial | 3.321928095 | 0.120869702 |
| conventional adjuvant chemotherapy | 3.321928095 | 0.120869702 |
| conventional chemotherapy | 3.321928095 | 0.120869702 |
| corticosteroids | 3.321928095 | 0.120869702 |
| cyclophosphamide | 3.032421478 | 1.67E-10 |
| cytoreduction | 3.321928095 | 0.120869702 |
| cytoreductive surgery | 3.906890596 | 0.013747874 |
| debulking surgery | 3.321928095 | 0.120869702 |
| diagnosis | 1.584962501 | 0.266781451 |
| differentiation | -2.807354922 | 1.07E-07 |
| disease | -0.415037499 | 0.850630219 |
| dna-damage | 1.736965594 | 0.422742778 |
| docetaxel | 3.321928095 | 0.120869702 |
| double-blind | 6.906890596 | 1.01E-25 |
| down-regulation | 3.321928095 | 0.120869702 |
| doxorubicin | 3.906890596 | 0.013747874 |
| drug-resistance | 4.321928095 | 0.001342727 |
| drugs | 1.736965594 | 0.422742778 |
| dysfunction | 3.906890596 | 0.013747874 |
| early breast cancer | 3.321928095 | 0.120869702 |
| efficacy | 3.129283017 | 0.000148872 |
| endometrial fibrosis | 1.736965594 | 0.422742778 |
| endometrial receptivity | 0.906890596 | 0.587922476 |
| endometrial regeneration | -0.765534746 | 0.679108938 |
| endometrium | -0.608809243 | 0.318177135 |
| endothelial growth-factor | 0.736965594 | 0.297539778 |
| endothelial progenitor cells | -0.263034406 | 1 |
| epithelial ovarian | 4.906890596 | 1.04E-05 |
| epithelial ovarian-cancer | 3.906890596 | 0.013747874 |
| epithelial-cells | -1.485426827 | 0.214846947 |
| erythropoietin | 3.321928095 | 0.120869702 |
| escalation | 3.321928095 | 0.120869702 |
| estrogen | -0.378511623 | 1 |
| estrogen therapy | 3.906890596 | 0.013747874 |
| exosome | -1.321928095 | 0.038293887 |
| exosomes | -0.963474124 | 0.266446526 |
| expression | -1.610957709 | 6.71E-05 |
| extracellular vesicle | -3.906890596 | 0.013747874 |
| extracellular vesicles | -3.64385619 | 0.000121203 |
| extremely preterm infants | 3.321928095 | 0.120869702 |
| factor vegf | 3.321928095 | 0.120869702 |
| failure | -0.201633861 | 1 |
| fallopian-tube | 3.906890596 | 0.013747874 |
| feasibility | 3.321928095 | 0.120869702 |
| fecal incontinence | 3.906890596 | 0.013747874 |
| fertility | 0.192645078 | 0.889520966 |
| fertility drugs | 3.321928095 | 0.120869702 |
| fertility preservation | -0.378511623 | 1 |
| fibrosis | 0.473931188 | 0.706449523 |
| follicles | 2 | 0.072558067 |
| follicular development | 3.321928095 | 0.120869702 |
| follow-up | 4.64385619 | 0.000121203 |
| g-csf | 3.906890596 | 0.013747874 |
| gene-expression | -2.201633861 | 0.029700187 |
| granulosa-cells | -0.137503524 | 1 |
| growth | 0 | 1 |
| growth factors | 3.321928095 | 0.120869702 |
| growth-factor | -0.321928095 | 0.866477063 |
| growth-factors | 0.736965594 | 0.873949692 |
| gynecologic-oncology-group | 5.129283017 | 8.75E-07 |
| hematopoietic rescue | 3.906890596 | 0.013747874 |
| hematopoietic stem cell transplantation | 3.321928095 | 0.120869702 |
| high-dose chemotherapy | 6.491853096 | 5.89E-19 |
| high-dose melphalan | 3.321928095 | 0.120869702 |
| hipec | 3.321928095 | 0.120869702 |
| hormone | 1 | 0.73457052 |
| hormone-replacement therapy | 3.321928095 | 0.120869702 |
| human amnion-derived mesenchymal stem cells (had-mscs) | 3.321928095 | 0.120869702 |
| human umbilical cord mesenchymal stem cells | 0.584962501 | 0.803032671 |
| human-papillomavirus | 3.321928095 | 0.120869702 |
| hyaluronic-acid | 3.321928095 | 0.120869702 |
| hyaluronic-acid gel | 4.64385619 | 0.000121203 |
| hypoxia | 0.321928095 | 1 |
| hypoxic-ischemic encephalopathy | 3.321928095 | 0.120869702 |
| hysteroscopic adhesiolysis | 5.321928095 | 7.17E-08 |
| hysteroscopy | 4.64385619 | 0.000121203 |
| identification | -1.519374159 | 0.09276585 |
| immunotherapy | 1.736965594 | 0.422742778 |
| impact | 0.321928095 | 1 |
| in vitro fertilization | 3.321928095 | 0.120869702 |
| infants | 3.058893689 | 0.002576341 |
| infertility | -0.874469118 | 0.196007973 |
| inflammation | -0.056583528 | 1 |
| inhaled nitric-oxide | 4.321928095 | 0.001342727 |
| inhibition | 4.64385619 | 0.000121203 |
| injection | 0.906890596 | 0.587922476 |
| injury | 0.38332864 | 0.736667246 |
| insights | 3.321928095 | 0.120869702 |
| insufficiency | 1.152003093 | 0.318426413 |
| intensification | 3.321928095 | 0.120869702 |
| intensity analysis | 3.321928095 | 0.120869702 |
| intensive chemotherapy | 3.906890596 | 0.013747874 |
| intensive therapy | 3.321928095 | 0.120869702 |
| intraperitoneal cisplatin | 3.906890596 | 0.013747874 |
| intrauterine adhesion | 2.974004791 | 1.76E-06 |
| intrauterine adhesions | 1.084888898 | 0.023656124 |
| intravenous cisplatin | 3.321928095 | 0.120869702 |
| in-vitro | -1.836501268 | 0.00033112 |
| in-vitro fertilization | 0.473931188 | 0.706449523 |
| irradiation | 1.321928095 | 0.58293887 |
| ischemia/reperfusion injury | 3.321928095 | 0.120869702 |
| ivf | 1.584962501 | 0.266781451 |
| late oligodendrocyte progenitors | 3.321928095 | 0.120869702 |
| live birth | 3.321928095 | 0.120869702 |
| long-term | -0.925999419 | 0.554700164 |
| long-term survival | 3.321928095 | 0.120869702 |
| macrophage | 3.321928095 | 0.120869702 |
| maintenance therapy | 5.129283017 | 8.75E-07 |
| management | 1.736965594 | 0.118250884 |
| mechanism | 1 | 0.73457052 |
| mechanisms | 0.222392421 | 0.881266699 |
| melphalan | 3.906890596 | 0.013747874 |
| membrane | 0.736965594 | 0.69709161 |
| menopause | 2 | 0.072558067 |
| menscs | 1.736965594 | 0.422742778 |
| mesenchymal stem cell | -0.963474124 | 0.266446526 |
| mesenchymal stem cells | -2.282933963 | 1.15E-05 |
| mesenchymal stem-cells | -0.273922722 | 0.441383084 |
| mesenchymal stromal cells | -2.510961919 | 0.008609633 |
| meta-analysis | 3.906890596 | 0.013747874 |
| metastasis | 1.736965594 | 0.422742778 |
| metastatic breast-cancer | 5.906890596 | 2.87E-12 |
| metastatic melanoma | 3.321928095 | 0.120869702 |
| metformin | 3.321928095 | 0.120869702 |
| micronuclei | 3.321928095 | 0.120869702 |
| migration | 0.447458977 | 0.668945874 |
| minimal residual disease | 3.321928095 | 0.120869702 |
| mirna | -3.321928095 | 0.007869702 |
| mitochondria | 1.736965594 | 0.422742778 |
| mitoxantrone | 3.321928095 | 0.120869702 |
| mmp-9 | 3.321928095 | 0.120869702 |
| mobilization | 1 | 0.73457052 |
| model | -0.847996907 | 0.272073913 |
| models | 1.736965594 | 0.422742778 |
| mortality | 3.321928095 | 0.120869702 |
| mouse | -2 | 0.057872264 |
| mouse model | -1.432959407 | 0.239217414 |
| mri | 3.321928095 | 0.120869702 |
| multicenter | 3.321928095 | 0.120869702 |
| multiple-myeloma | 3.321928095 | 0.120869702 |
| multivariate-analysis | 3.321928095 | 0.120869702 |
| mutations | 1.321928095 | 0.58293887 |
| neoadjuvant chemotherapy | 4.321928095 | 0.001342727 |
| neoplasia | 3.321928095 | 0.120869702 |
| newborn | 3.321928095 | 0.120869702 |
| nf-kappa-b | 1.099535674 | 0.477659735 |
| nitric oxide | 3.321928095 | 0.120869702 |
| non-hodgkins-lymphoma | 4.321928095 | 0.001342727 |
| open-label | 4.64385619 | 0.000121203 |
| operative hysteroscopy | 3.321928095 | 0.120869702 |
| outcomes | 3.321928095 | 0.120869702 |
| ovarian cancer | 7.17990909 | 2.06E-31 |
| ovarian cancer stem cells | 3.321928095 | 0.120869702 |
| ovarian hyperstimulation syndrome | 3.321928095 | 0.120869702 |
| ovarian neoplasm | 3.321928095 | 0.120869702 |
| ovarian-cancer | 7.17990909 | 2.06E-31 |
| ovarian-cancer risk | 3.321928095 | 0.120869702 |
| ovary | -0.137503524 | 1 |
| ovary cancer | 3.321928095 | 0.120869702 |
| ovulation induction | 4.321928095 | 0.001342727 |
| oxidative stress | -0.807354922 | 0.382307823 |
| paclitaxel | 6.845490051 | 1.37E-24 |
| pancreatic-cancer | 3.321928095 | 0.120869702 |
| parp inhibitor | 3.321928095 | 0.120869702 |
| pathogenesis | -1.070389328 | 0.451295199 |
| pathway | 1.906890596 | 0.175032934 |
| pathways | 3.906890596 | 0.013747874 |
| pd-l1 expression | 3.321928095 | 0.120869702 |
| pegylated liposomal doxorubicin | 4.321928095 | 0.001342727 |
| pelvic floor disorders | 4.321928095 | 0.001342727 |
| perinatal asphyxia | 3.321928095 | 0.120869702 |
| peripheral blood stem cell transplantation | 3.321928095 | 0.120869702 |
| peripheral stem cell transplantation | 3.321928095 | 0.120869702 |
| peripheral-blood | -1.678071905 | 0.054868765 |
| phase-i trial | 3.906890596 | 0.013747874 |
| phase-ii | 4.906890596 | 1.04E-05 |
| phase-ii trial | 5.64385619 | 4.63E-10 |
| phase-iii | 4.321928095 | 0.001342727 |
| phase-iii trial | 4.321928095 | 0.001342727 |
| pi3k | 3.321928095 | 0.120869702 |
| placebo | 3.906890596 | 0.013747874 |
| platelet-rich plasma | 0.836501268 | 0.40803376 |
| platelet-rich plasma (prp) | 3.321928095 | 0.120869702 |
| platinum | 3.321928095 | 0.120869702 |
| platinum-resistant | 3.321928095 | 0.120869702 |
| polyphenols | 3.321928095 | 0.120869702 |
| postmenopausal women | 1.321928095 | 0.58293887 |
| preeclampsia | -1.232660757 | 0.124555257 |
| pregnancy | -1.495695163 | 0.000613881 |
| premature ovarian failure | -2.944858446 | 0.000863093 |
| premature ovarian insufficiency | 2.206450877 | 6.98E-05 |
| premature ovarian insufficiency (poi) | 3.906890596 | 0.013747874 |
| prematurity | 3.321928095 | 0.120869702 |
| prevalence | 5.129283017 | 8.75E-07 |
| prevention | 2.584962501 | 6.10E-06 |
| primary ovarian insufficiency | 1.584962501 | 0.266781451 |
| primary peritoneal | 3.321928095 | 0.120869702 |
| progenitor cells | -2.232660757 | 0.000816812 |
| prognosis | 3.321928095 | 0.120869702 |
| progression | 3.321928095 | 0.120869702 |
| proliferation | -2.765534746 | 0.002449074 |
| prospective randomized trial | 3.321928095 | 0.120869702 |
| prospective randomized-trial | 3.321928095 | 0.120869702 |
| protein | 0.321928095 | 1 |
| prp | 3.906890596 | 0.013747874 |
| quality | 3.321928095 | 0.120869702 |
| quality-of-life | 2.321928095 | 0.099498234 |
| radiotherapy | 3.321928095 | 0.120869702 |
| randomized controlled-trial | 3.906890596 | 4.19E-07 |
| randomized phase-2 | 3.321928095 | 0.120869702 |
| randomized phase-iii | 4.64385619 | 0.000121203 |
| randomized trial | 6.22881869 | 1.33E-15 |
| randomized-trial | 6.569855608 | 4.44E-20 |
| rat | -0.584962501 | 0.827052358 |
| rat model | 0 | 1 |
| rats | 0.736965594 | 0.873949692 |
| receptor | -0.180572246 | 1 |
| recombinant-human-erythropoietin | 3.321928095 | 0.120869702 |
| recurrence | 3.906890596 | 0.013747874 |
| recurrent | 3.906890596 | 0.013747874 |
| recurrent epithelial ovarian | 3.321928095 | 0.120869702 |
| reformation | 3.906890596 | 0.013747874 |
| refractory ovarian | 3.321928095 | 0.120869702 |
| regeneration | -0.514573173 | 0.466413527 |
| regenerative medicine | -1.30256277 | 0.171791362 |
| regimens | 3.321928095 | 0.120869702 |
| repair | -0.847996907 | 0.456750402 |
| rescue | 3.906890596 | 0.013747874 |
| resistance | 4.906890596 | 1.04E-05 |
| respiratory-distress-syndrome | 4.321928095 | 0.001342727 |
| responses | 1 | 0.73457052 |
| rhoa | 3.321928095 | 0.120869702 |
| risk-factors | 2.584962501 | 0.002880885 |
| safety | 1.321928095 | 0.58293887 |
| salvage treatment | 3.906890596 | 0.013747874 |
| senescence | -0.263034406 | 1 |
| signaling pathway | 0.152003093 | 1 |
| skeletal-muscle | 3.321928095 | 0.120869702 |
| soft-tissue sarcoma | 3.321928095 | 0.120869702 |
| solid tumors | 4.64385619 | 0.000121203 |
| stage-iii | 6.569855608 | 4.44E-20 |
| stem cell migration | 3.321928095 | 0.120869702 |
| stem cell therapy | -1.378511623 | 0.266246741 |
| stem cell transplantation | 0.943416472 | 0.339133126 |
| stem cells | -1.047305715 | 0.037239155 |
| stem-cell support | 4.64385619 | 0.000121203 |
| stem-cell transplantation | 3.736965594 | 4.11E-06 |
| stem-cells | 0.33198176 | 0.217912586 |
| stimulation | 1.584962501 | 0.266781451 |
| stress urinary incontinence | 3.321928095 | 0.120869702 |
| stress urinary-incontinence | 3.321928095 | 0.120869702 |
| stromal cells | -2.015941544 | 2.26E-06 |
| support | 3.321928095 | 0.120869702 |
| survival | 2.852442812 | 3.39E-07 |
| systematic review | 3.906890596 | 0.013747874 |
| target | 1.736965594 | 0.422742778 |
| t-cells | -0.925999419 | 0.554700164 |
| therapy | 0.701776166 | 0.02836072 |
| thickness | 0.321928095 | 1 |
| thin endometrium | 0.447458977 | 0.668945874 |
| tissue | -0.519374159 | 0.514185824 |
| tissue engineering | 0.906890596 | 0.587922476 |
| toxicity | 1.584962501 | 0.266781451 |
| transplantation | -1 | 0.003514603 |
| treatment | 5.129283017 | 8.75E-07 |
| trial | 3.321928095 | 0.120869702 |
| tumor-growth | 1.321928095 | 0.58293887 |
| tumors | 3.321928095 | 0.120869702 |
| umbilical cord mesenchymal stem cells | -0.137503524 | 1 |
| uterus | -1.137503524 | 0.406581188 |
| vitamin-a supplementation | 3.321928095 | 0.120869702 |
| volume-targeted ventilation | 3.321928095 | 0.120869702 |
| women | 0.321928095 | 0.568431816 |

**Supplementary Table S4. Detailed data for HRCS score**

| **Title** | **Research area** | **HRCS score** |
| --- | --- | --- |
| Regenerative potential of different extracellular vesicle subpopulations derived from clonal mesenchymal stem cells in a mouse model of chemotherapy-induced premature ovarian failure | hucMSC-exos miR-146a-5p, POF | 4 |
| Homogenous subpopulation of human mesenchymal stem cells and their extracellular vesicles restore function of endometrium in an experimental rat model of Asherman syndrome | cMSCs hMSCs, IUA | 4 |
| Clusterin-carrying extracellular vesicles derived from human umbilical cord mesenchymal stem cells restore the ovarian function of premature ovarian failure mice through activating the PI3K/AKT pathway | Extracellular vesicles，Premature ovarian failure (POF),UC-MSCs | 4 |
| Therapeutic effects of human umbilical cord mesenchymal stem cell-derived extracellular vesicles on ovarian functions through the PI3K/Akt cascade in mice with premature ovarian failure | hucMSC-EVs,PI3K/Akt pathway,Premature ovarian failure | 4 |
| Transplantation of the LRP1high subpopulation of human umbilical cord-derived mesenchymal stem cells improves ovarian function in mice with premature ovarian failure and aged mice | Ovarian aging ,Premature ovarian failure | 4 |
| Human umbilical cord-derived mesenchymal stem cells (hUC-MSCs) alleviate excessive autophagy of ovarian granular cells through VEGFA/PI3K/AKT/mTOR pathway in premature ovarian failure rat model | hUC-MSCs,Premature ovarian failure | 4 |
| Human umbilical cord mesenchymal stem cell-derived extracellular vesicles harboring IGF-1 improve ovarian function of mice with premature ovarian insufficiency through the Nrf2/HO-1 pathway | hUC-MSCs-EVs Premature ovarian insufficiency | 4 |
| Human urine stem cells protect against cyclophosphamide-induced premature ovarian failure by inhibiting SLC1A4-mediated outflux of intracellular serine in ovarian granulosa cells | Human urine stem cells,Premature ovarian failure | 4 |
| Beneficial effects of human umbilical cord mesenchymal stem cell (HUCMSC) transplantation on cyclophosphamide (CTX)-induced premature ovarian failure (POF) in Tibetan miniature pigs | Human umbilical cord MSCs,Premature ovarian failure | 4 |
| Adipose-derived stem cells promote the repair of chemotherapy-induced premature ovarian failure by inhibiting granulosa cells apoptosis and senescence | Adipose-derived stem cells Premature ovarian failure | 4 |
| 3D hUC-MSC spheroids exhibit superior resistance to autophagy and apoptosis of granulosa cells in POF rat model | 3D hUC-MSC spheroids Premature ovarian failure | 4 |
| Exosomes from adipose-derived stem cells alleviate premature ovarian failure via blockage of autophagy and AMPK/mTOR pathway | Adipose-derived stem cell exosomes AMPK/mTOR pathway Premature ovarian failure | 4 |
| Improving Granulosa Cell Function in Premature Ovarian Failure with Umbilical Cord Mesenchymal Stromal Cell Exosome-Derived hsa_circ_0002021 | HUMSC-derived exosomes Premature ovarian failure | 4 |
| Human hair follicle-derived mesenchymal stem cells improve ovarian function in cyclophosphamide-induced POF mice | Hair follicle-derived MSCs KEAP1/NRF2/HO-1 Premature ovarian failure | 4 |
| The Effect of Human Umbilical Cord Mesenchymal Stem Cell on Premature Ovarian Cell Senilism Through miR-10a | miR-10a-modified exosomes Premature ovarian failure | 4 |
| Exosomes From Human Umbilical Cord Mesenchymal Stem Cells Alleviate Oxidative Stress-Induced POI by Regulating Autophagic Homeostasis Through the AMPK Pathway | Exosome therapy Premature ovarian insufficiency | 4 |
| Bone marrow mesenchymal stem cells expressing Neat-1, Hotair-1, miR-21, miR-644, and miR-144 subsided cyclophosphamide-induced ovarian insufficiency by remodeling the IGF-1-kisspeptin system, ovarian apoptosis, and angiogenesis | BM-MSCs Ovarian insufficiency | 4 |
| Reduction of Apoptotic Gene Expression by Platelet-rich Plasma in a Mouse Model of Premature Ovarian Failure | Platelet-rich plasma Premature ovarian failure | 4 |
| EIF4A3-Induced Exosomal circLRRC8A Alleviates Granulosa Cells Senescence Via the miR-125a-3p/NFE2L1 axis | circLRRC8A Granulosa cell senescence | 4 |
| Endometrial stem cells alleviate cisplatin-induced ferroptosis of granulosa cells by regulating Nrf2 expression | Endometrial stem cells Granulosa cell ferroptosis | 4 |
| 3D cultured human umbilical cord mesenchymal stem cell spheroids regulate oxidative stress and iron homeostasis through the Nrf2 pathway to resist ferroptosis in ovarian granulosa cells and ovarian dysfunction | 3D hUCMSC spheroids Granulosa cell ferroptosis | 4 |
| Menstrual Blood-Derived Endometrial Stem Cells Ameliorate Ovarian Senescence by Relieving Oxidative Stress-Induced Inflammation | Menstrual blood-derived endometrial stem cells Ovarian senescence Oxidative stress and inflammation | 4 |
| Microfluidic Encapsulation of Exosomes Derived from Lipopolysaccharide-Treated Mesenchymal Stem Cells in Hyaluronic Acid Methacryloyl to Restore Ovarian Function in Mice | Microfluidic encapsulated exosomes POF therapy | 4 |
| Human embryonic stem cell-derived immunity-and-matrix-regulatory cells promote endometrial repair and fertility restoration in IUA rats | Endometrial fibrosis Immunity-and-matrix-regulatory cells Intrauterine adhesion | 4 |
| Comparison of the therapeutic effects between stem cells and exosomes in primary ovarian insufficiency: as promising as cells but different persistency and dosage | POI therapy | 4 |
| The protective mechanism of human umbilical cord mesenchymal stem cell-derived exosomes against neutrophil extracellular trap-induced placental damage | hUCMSC-derived exosomes Preeclampsia | 4 |
| Effect of preconditioning of human umbilical cord mesenchymal stem cells with hydrogen peroxide on the therapeutic potential of MSCs in the cyclophosphamide-induced premature ovarian failure mice model | H₂O₂ preconditioning Human umbilical cord MSCs, hUCV-MSCs | 4 |
| Fertility protection: a novel approach using pretreatment with mesenchymal stem cell exosomes to prevent chemotherapy-induced ovarian damage in a mouse model | Exosome pretreatment for fertility protection Prevention of chemotherapy-induced ovarian damage | 4 |
| Exosomes derived from mesenchymal stem cells attenuate NLRP3-related pyroptosis in autoimmune premature ovarian insuf fi ciency via the NF- k B pathway | Autoimmune premature ovarian insufficiency MSC-derived exosomes | 4 |
| Pyrroloquinoline quinone promotes human mesenchymal stem cell-derived mitochondria to improve premature ovarian insufficiency in mice through the SIRT1/ATM/p53 pathway | Mitochondrial transfer Pyrroloquinoline quinone | 4 |
| Menstrual Blood-Derived Mesenchymal Stem Cells Improve Endometrial Receptivity in a Mouse Model of Embryonic Implantation Dysfunction | Endometrial receptivity Menstrual blood-derived MSCs | 4 |
| VitroGel-loaded human MenSCs promote endometrial regeneration and fertility restoration | VitroGel-loaded human MenSCs | 4 |
| Enhanced myofibroblast differentiation of eMSCs in intrauterine adhesions | eMSCs, IUA | 2 |
| The mechanism of adipose mesenchymal stem cells to stabilize the immune microenvironment of pelvic floor injury by regulating pyroptosis and promoting tissue repair | Adipose mesenchymal stem cells Immune microenvironment Pelvic organ prolapse Pyroptosis Tissue repair | 4 |
| Fertility restoration in mice with chemotherapy induced ovarian failure using differentiated iPSCs | Fertility restoration Induced pluripotent stem cells Ovarian failure | 4 |
| FtMt reduces oxidative stress-induced trophoblast cell dysfunction via the HIF-1/VEGF signaling pathway | HIF-1α/VEGF signaling pathway Mitochondrial ferritin (FtMt) Oxidative stress Preeclampsia (PE) Trophoblast cells | 4 |
| HuMenSCs Initiate the Uterus Stromal Decidualization in Mouse | Decidualization Human menstrual blood stem cells Uterine stroma | 4 |
| Ovarian Function and Spontaneous Pregnancy After Hematopoietic Stem Cell Transplantation for Leukemia Before Puberty: An LEA Cohort Study | Female ovarian function Hematopoietic stem cell transplantation Leukemia Prepuberty Spontaneous pregnancy | 3 |
| A 16-year bicentric retrospective analysis of ovarian tissue cryopreservation in pediatric units: indications, results, and outcome | Ovarian tissue cryopreservation Pediatric patients | 3 |
| Umbilical cord mesenchymal stem cells from gestational diabetes show impaired ability to up-regulate paracellular permeability from sub-endothelial niche | Gestational diabetes mellitus Paracellular permeability Umbilical cord mesenchymal stem cells Vascular endothelial barrier. | 4 |
| The Effect of Normal Follicular Fluid on the Differentiation of PCOS Ovarian Stem Cells into Oocyte-Like Cells | Normal follicular fluid Oocyte-like cell differentiation Ovarian stem cells Polycystic ovarian syndrome | 4 |
| Improvement of Inflammation and Abnormal Vascularization by TSP1 Treatment Combined with ADSCs Transplantation in Mice with Induced Polycystic Ovary Syndrome | Adipose-derived mesenchymal stem cells Polycystic ovary syndrome Thrombospondin 1 | 4 |
| BMSCs-EVs Alleviate Pelvic Floor Dysfunction in Mice by Reducing Inflammation and Promoting Tissue Regeneration | BMSC-derived extracellular vesicles Pelvic floor dysfunction Tissue regeneration | 4 |
| Human endometrium-derived mesenchymal stem/stromal cells application in endometrial-factor induced infertility | Endometrial repair Endometrial-factor induced infertility Human endometrium-derived mesenchymal stem/stromal cells | 4 |
| Exosomes secreted from adipose-derived stem cells inhibit M1 macrophage polarization ameliorate chronic endometritis by regulating SIRT2/NLRP3 | Adipose-derived stem cell exosomes Chronic endometritis Macrophage polarization | 4 |
| 3D bio-printed endometrial construct restores the full-thickness morphology and fertility of injured uterine endometrium | 3D bio-printed endometrial construct Endometrial repair Fertility restoration | 4 |
| Administration of adipose-derived mesenchymal stem cell conditioned medium improves ovarian function in polycystic ovary syndrome rats: involvement of epigenetic modifiers system | Adipose-derived mesenchymal stem cell conditioned medium | 4 |
| Improved uterine conditions following ovarian transplantation of adipose-derived mesenchymal stem cell conditioned media in rats with letrozole-induced polycystic ovary syndrome: Histomorphometrical, biochemical, and molecular analysis | Adipose-derived mesenchymal stem cell conditioned media Polycystic ovary syndrome Uterine condition improvement | 4 |
| Effects of human placenta cryopreservation on molecular characteristics of placental mesenchymal stromal cells | Human placenta cryopreservation Molecular characteristics Placental mesenchymal stromal cells | 4 |
| The Protective Effect of Adipose-Derived Stromal Vascular Fraction on Ovarian Function in Rats with Cyclophosphamide-Induced Ovarian Damage | Adipose-derived stromal vascular fraction Ovarian function protection | 4 |
| Autologous non-invasively derived stem cells mitochondria transfer shows therapeutic advantages in human embryo quality rescue | Autologous stem cell mitochondria transfer Embryo quality rescue | 4 |
| Endometrial stromal cells from women with repeated implantation failure display impaired invasion towards trophoblastic spheroids | nvasive ability Endometrial stromal cells | 4 |
| The improvement of inflammatory infiltration and pregnancy outcome in mice with recurrent spontaneous abortion by human amniotic mesenchymal stem cells | Human amniotic mesenchymal stem cells inflammatory infiltration pregnancy outcome recurrent spontaneous abortion | 4 |
| A novel therapeutic approach for endometriosis using adipose-derived stem cell-derived conditioned medium- A new hope for endometriotic patients in improving fertility | Adipose-derived stem cell-derived conditioned medium endometriosis fertility | 4 |
| Bone marrow mesenchymal stem cell-derived exosomes shuttle microRNAs to endometrial stromal fibroblasts that promote tissue proliferation /regeneration/ and inhibit differentiation | Bone marrow mesenchymal stem cell-derived exosomes endometrial stromal fibroblasts microRNAs tissue regeneration | 4 |
| Exosomal miR-146a-5p derived from bone marrow mesenchymal stromal cells regulate Th1/Th2 balance and alleviates immune thrombocytopenia in pregnancy | Bone marrow mesenchymal stromal cell exosomes immune thrombocytopenia in pregnancy miR-146a-5p Th1/Th2 balance | 4 |
| Umbilical cord mesenchymal stem cells restore ovarian function and suppress apoptosis in a rat model of chemotherapy-induced premature ovarian insufficiency | Premature ovarian failure stem cells | 4 |
| Photo-Cross-linked Gelatin Methacryloyl Hydrogels Enable the Growth of Primary Human Endometrial Stromal Cells and Epithelial Gland Organoids | 3D model endometrial cells Photo-cross-linked gelatin methacryloyl hydrogels reproductive health | 3 |
| Low dose acetyl salicylic acid (LDA) mediates epigenetic changes in preeclampsia placental mesenchymal stem cells similar to cells from healthy pregnancy | epigenetics placental mesenchymal stem cells preeclampsia | 4 |
| Small RNA sequencing of exosomal microRNAs reveals differential expression of microRNAs in preeclampsia | biomarkers Exosomal microRNAs preeclampsia | 3 |
| Combined Application of Exosomes and FPR2 Agonist LXA4 in Controlling Fetal Membrane Inflammation and Promoting Fetal Membrane Tissue Repair | Exosomes fetal membrane inflammation FPR2 agonist LXA4 tissue repair | 4 |
| Targeting Decidual CD16+ Immune Cells with Exosome-Based Glucocorticoid Nanoparticles for Miscarriage | Exosomes GC - Exo - CD16Ab immune regulation miscarriage | 4 |
| The Effects of the Follicle-Stimulating Hormone on Human Follicular Fluid-Derived Stromal Cells | female infertility Follicle-Stimulating Hormone follicular fluid-derived stromal cells | 2 |
| hUC-MSC Combined with DHEA Alleviates Ovarian Senescence in Naturally Aging Mice through Enhancing Antioxidant Capacity and Inhibiting Inflammatory Response | DHEA ovarian aging UC-MSCs | 4 |
| The effect of BMSCs on implantation mechanisms and pregnancy process in the experimental Asherman model | Asherman syndrome BMSCs | 4 |
| Effects of mesenchymal stem cells and heparan sulfate mimetics on urethral function and vaginal wall biomechanics in a simulated rat childbirth injury model | Pelvic floor disorders | 4 |
| Huc-MSC-derived exosomal miR-144 alleviates inflammation in LPS-induced preeclampsia-like pregnant rats via the FosB/ Flt-1 pathway | Preeclampsia | 4 |
| Circ_0008440 Inhibits Proliferation and Promotes Apoptosis of Trophoblast Cells through the miR-194-5p/PFKFB2 Axis | Preeclampsia | 1 |
| Intrauterine adhesions repair with menstrual blood-derived mesenchymal stem cells via CXCL13-CXCR5 signal axis and its mechanism | Intrauterine Adhesions | 4 |
| A Novel Method to Repair Thin Endometrium and Restore Fertility Based on Menstruation-Derived Stem Cell | Thin endometrium | 4 |
| Hypoxic Preconditioning Enhances Cellular Viability and Migratory Ability: Role of DANCR/miR-656-3p/HIF-1α Axis in Placental Mesenchymal Stem Cells | Preeclampsia | 1 |
| Melatonin-pretreated human umbilical cord mesenchymal stem cells improved endometrium regeneration and fertility recovery through macrophage immunomodulation in rats with intrauterine adhesions | Intrauterine adhesions | 4 |
| Low-intensity pulsed ultrasound combined with microbubbles enhances stem cell-based therapy for endometrial injury and intrauterine adhesion | intrauterine adhesions | 4 |
| Intrauterine injection of bioengineered hydrogel loaded exosomes derived from HUCM stem cells and spermidine prominently augments the pregnancy rate in thin endometrium rats | thin endometrium | 4 |
| Minimally invasive delivery of human umbilical cord-derived mesenchymal stem cells by an injectable hydrogel via Diels-Alder click reaction for the treatment of intrauterine adhesions | Intrauterine adhesions | 4 |
| Exosome-Based Regimen Rescues Endometrial Fibrosis in Intrauterine Adhesions Via Targeting Clinical Fibrosis Biomarkers | Intrauterine adhesions | 4 |
| Long-term therapeutic effects of allogeneic mesenchymal stem cell transplantation for intrauterine adhesions | Intrauterine adhesion | 4 |
| Secretion of WNT7A by UC-MSCs assist in promoting the endometrial epithelial regeneration | Stem cell therapy for intrauterine adhesions | 1 |
| A construct of adipose-derived mesenchymal stem cells-laden collagen scaffold for fertility restoration by inhibiting fibrosis in a rat model of endometrial injury | Severe endometrium damage | 4 |
| Telocyte-Derived Exosomes Provide an Important Source of Wnts That Inhibits Fibrosis and Supports Regeneration and Repair of Endometrium | Intrauterine adhesions | 4 |
| A smart therapeutic approach for improving thin endometrium in rodent models - A maiden intra uterine preclinical approach | Recurrent endometrial injuries | 4 |
| Transplantation of human endometrial perivascular stem cells with hydroxy saffron yellow A promotes uterine repair in rats | Intrauterine adhesions | 4 |
| Novel injectable sodium alginate hydrogel developed for improved endometrial repair with human umbilical cord mesenchymal stem cells | endometrial repair | 4 |
| Adipocyte alterations in endometriosis: reduced numbers of stem cells and microRNA induced alterations in adipocyte metabolic gene expression | Endometriosis | 4 |
| Human amniotic epithelial cells improve uterine spiral artery remodeling to ameliorate preeclampsia in a rat model | Preeclampsia | 4 |
| Effect of collagen endometrial patch loaded with adipose-derived mesenchymal stem cells on endometrial regeneration in rats with a thin endometrium | thin endometrium | 4 |
| Human umbilical cord mesenchymal stem cells regulate glutathione metabolism depending on the ERK-Nrf2-HO-1 signal pathway to repair phosphoramide mustard-induced ovarian cancer cells | human ovarian cancer | 4 |
| Mesenchymal stem cells promote ovarian reconstruction in mice | Ovrian reconstruction | 4 |
| Effects of Adipose-tissue Derived Mesenchymal Stem Cells on PBMCs in Co-culture with HeLa Cell Line | Cervical cancer | 4 |
| Effective repair of endometrial injury in rats using enzyme cross-linked gelatin hydrogels with human menstrual blood-derived stem cells | endometrial injury | 4 |
| Transplantation of endothelial progenitor cells for improving placental perfusion in preeclamptic rats | preeclampsia | 4 |
| Cigarette smoke inhibits recruitment of bone-marrow-derived stem cells to the uterus | Cigarette smoking | 2 |
| A Robust and Highly Efficient Approach for Isolation of Mesenchymal Stem Cells From Wharton's Jelly for Tissue Repair | endometrial injury | 4 |
| Uterine Infusion With Bone Marrow Mesenchymal Stem Cells Improves Endometrium Thickness in a Rat Model of Thin Endometrium | thin endometrium | 4 |
| Endometrial stem cells repair injured endometrium and induce angiogenesis via AKT and ERK pathways | Intrauterine adhesions | 4 |
| Transplantation induced pluripotent stem cells in situ improves fertility outcome impaired by intrauterine adhesions in mice | Intrauterine adhesion | 5 |
| Therapeutic benefit of mesenchymal stem cells in pregnant rats with angiotensin receptor agonistic autoantibody-induced hypertension: Implications for immunomodulation and cytoprotection | Preeclampsia | 4 |
| Differentiation of primordial germ cells from premature ovarian insufficiency-derived induced pluripotent stem cells | Premature ovarian insufficiency | 1 |
| Menstrual blood CD146+ mesenchymal stem cells reduced fibrosis rate in the rat model of premature ovarian failure | premature ovarian failure | 4 |
| The synergistic effect of electroacupuncture and bone mesenchymal stem cell transplantation on repairing thin endometrial injury in rats | thin endometrial injury | 5 |
| EphA2-positive human umbilical cord-derived mesenchymal stem cells exert anti-fibrosis and immunomodulatory activities via secretion of prostaglandin E2 | EphA2 | 1 |
| Decidualization Potency and Epigenetic Changes in Human Endometrial Origin Stem Cells During Propagation | hEndSCs | 1 |
| The effects of human amnion membrane-derived mesenchymal stem cells conditioned medium on ionizing radiation-induced premature ovarian failure and endoplasmic reticulum stress-related apoptosis mechanism | Premature ovarian failure | 2 |
| Impact of administering umbilical cord-derived mesenchymal stem cells to cynomolgus monkeys with endometriosis | endometriosis | 4 |
| Regenerative therapy by endometrial mesenchymal stem cells in thin endometrium with repeated implantation failure. A novel strategy | Thin endometrium | 4 |
| Effects of human umbilical cord mesenchymal stem cells on intrauterine adhesions in a rat model | intrauterine adhesions | 4 |
| Bone-marrow-derived endothelial progenitor cells contribute to vasculogenesis of pregnant mouse uterus | EPCs | 2 |
| The restorative effects of adipose-derived mesenchymal stem cells on damaged ovarian function | damaged ovarian | 4 |
| Mesenchymal stromal cell-derived extracellular vesicle therapy prevents preeclamptic physiology through intrauterine immunomodulation | Preeclampsia | 5 |
| Intra-ovarian injection of bone marrow-derived c-Kit+ cells for ovarian rejuvenation in menopausal rats | premature ovarian failure | 5 |
| Ability of human umbilical cord mesenchymal stem cells to repair chemotherapy-induced premature ovarian failure | Premature ovarian insufficiency (POI) and premature ovarian failure (POF) | 5 |
| Adipose-derived stem cells transplantation improves endometrial injury repair | intrauterine adhesion | 4 |
| Mesenchymal stem cell therapy attenuates complement C3 deposition and improves the delicate equilibrium between angiogenic and anti-angiogenic factors in abortion-prone mice | recurrent spontaneous abortions | 4 |
| The Dose-Related Efficacy of Human Placenta-Derived Mesenchymal Stem Cell Transplantation on Antioxidant Effects in a Rat Model with Ovariectomy | Oxidative stress | 4 |
| Placenta-Derived Mesenchymal Stem Cells Restore the Ovary Function in an Ovariectomized Rat Model via an Antioxidant Effect | Oxidative stress | 4 |
| Human adipose-derived stromal cells transplantation prolongs reproductive lifespan on mouse models of mild and severe premature ovarian insufficiency | Premature Ovarian Insufficiency | 4 |
| Exploration of eMSCs with HA-GEL system in repairing damaged endometrium after endometrial cancer with fertility-sparing treatment | endometrial cancer | 5 |
| Conservative Hypomethylation of Mesenchymal Stem Cells and Their Secretome Restored the Follicular Development in Cisplatin-Induced Premature Ovarian Failure Mice | Premature ovarian failure | 4 |
| Deciphering decidual deficiencies in recurrent spontaneous abortion and the therapeutic potential of mesenchymal stem cells at single-cell resolution | Recurrent spontaneous abortion | 1 |
| Progesterone regulates gut microbiota mediating bone marrow mesenchymal stem cell injury in immune thrombocytopenia patients during pregnancy | Immune thrombocytopenia during pregnancy | 2 |
| Synergistic effect of Huyang Yangkun Formula and embryonic stem cells on 4-vinylcyclohexene diepoxide induced premature ovarian insufficiency in mice | premature ovarian insufficiency | 5 |
| Perivascular Stem Cell-Derived Cyclophilin A Improves Uterine Environment with Asherman's Syndrome via HIF1伪-Dependent Angiogenesis | Asherman's syndrome | 1 |
| Effects of single and multiple transplantations of human umbilical cord mesenchymal stem cells on the recovery of ovarian function in the treatment of premature ovarian failure in mice | premature ovarian failure | 5 |
| Human embryonic stem cell-derived mesenchymal stem cells improved premature ovarian failure | Premature ovarian failur | 4 |
| Human Umbilical Cord Mesenchymal Stem Cells Improve Ovarian Function in Chemotherapy-Induced Premature Ovarian Failure Mice Through Inhibiting Apoptosis and Inflammation via a Paracrine Mechanism | premature ovarian failure | 4 |
| Human Umbilical Cord Mesenchymal Stem Cell-Derived Exosomes Improve Ovarian Function and Proliferation of Premature Ovarian Insufficiency by Regulating the Hippo Signaling Pathway | Premature ovarian insufficiency | 4 |
| The Effects of Human Umbilical Cord Mesenchymal Stem Cell Transplantation on Female Fertility Restoration in Mice | Female fertility | 4 |
| Matrigel/Umbilical Cord-Derived Mesenchymal Stem Cells Promote Granulosa Cell Proliferation and Ovarian Vascularization in a Mouse Model of Premature Ovarian Failure | Premature Ovarian Failure | 4 |
| Fibroblast Growth Factor 7 Regulates Proliferation and Decidualization of Human Endometrial Stromal Cells via ERK and JNK Pathway in an Autocrine Manner | Proliferation and Decidualization of Human Endometrial Stromal Cells | 2 |
| Human Umbilical Cord Mesenchymal Stem Cells Encapsulated with Pluronic F-127 Enhance the Regeneration and Angiogenesis of Thin Endometrium in Rat via Local IL-1β Stimulation | thin endometrium | 4 |
| TLR4 Modulates Senescence and Paracrine Action in Placental Mesenchymal Stem Cells via Inhibiting Hedgehog Signaling Pathway in Preeclampsia | Preeclampsia | 4 |
| Decidual mesenchymal stem/stromal cell-derived extracellular vesicles ameliorate endothelial cell proliferation, inflammation, and oxidative stress in a cell culture model of preeclampsia | Preeclampsia | 4 |
| Human umbilical cord mesenchymal stem cells restore the ovarian metabolome and rescue premature ovarian insufficiency in mice | Premature ovarian insufficiency | 4 |
| Exosomes Derived from Adipose Mesenchymal Stem Cells Restore Functional Endometrium in a Rat Model of Intrauterine Adhesions | Intrauterine adhesion | 4 |
| Unresponsive thin endometrium caused by Asherman syndrome treated with umbilical cord mesenchymal stem cells on collagen scaffolds: a pilot study | Asherman syndrome/ thin endometrium | 5 |
| Human umbilical cord mesenchymal stem cells (hUCMSCs) promotes the recovery of ovarian function in a rat model of premature ovarian failure (POF) | Premature Ovarian Failure | 4 |
| Platelet-rich plasma improves therapeutic effects of menstrual blood-derived stromal cells in rat model of intrauterine adhesion | Intrauterine adhesion | 4 |
| Concentrated exosomes from menstrual blood-derived stromal cells improves ovarian activity in a rat model of premature ovarian insufficiency | Premature ovarian insufficiency | 4 |
| Concentrated small extracellular vesicles from menstrual blood-derived stromal cells improve intrauterine adhesion, a pre-clinical study in a rat model | Intrauterine adhesion | 4 |
| Intrauterine Injection of Umbilical Cord Mesenchymal Stem Cell Exosome Gel Significantly Improves the Pregnancy Rate in Thin Endometrium Rats | thin endometrium | 4 |
| Umbilical Cord Mesenchymal Stem Cells Ameliorate Premature Ovarian Insufficiency in Rats | Premature ovarian insufficiency | 4 |
| Comparison of CD146+/- mesenchymal stem cells in improving premature ovarian failure | Premature Ovarian Failure | 4 |
| Transplantation of umbilical cord-derived mesenchymal stem cells promotes the recovery of thin endometrium in rats | thin endometrium | 4 |
| The protective effects of human umbilical cord mesenchymal stem cell-derived extracellular vesicles on cisplatin-damaged granulosa cells | cisplatin (CDDP)-damaged granulosa cells (GCs) in vitro | 4 |
| Organoid Transplantation Can Improve Reproductive Prognosis by Promoting Endometrial Repair in Mice | Intrauterine adhesion | 4 |
| Multi-Lineage Human Endometrial Organoids on Acellular Amniotic Membrane for Endometrium Regeneration | Asherman's syndrome | 4 |
| Mesenchymal stem cells enhance Treg immunosuppressive function at the fetal-maternal interface | recurrent spontaneous abortion (RSA) | 4 |
| Intra-ovarian injection of autologous menstrual blood-derived-mesenchymal stromal cells: a safe and promising method to improve pregnancy rate in poor ovarian responders | Poor ovarian response (POR) | 5 |
| Extracellular Vesicles Derived from Human Umbilical Cord MSC Improve Vascular Endothelial Function in In Vitro and In Vivo Models of Preeclampsia through Activating Arginine Metabolism | Preeclampsia | 4 |
| Intrauterine Infusion and Hysteroscopic Injection of Autologous Platelet-Rich Plasma for Patients with a Persistent Thin Endometrium: A Prospective Case-Control Study | thin endometrium | 5 |
| Bone Marrow Mesenchymal Stem Cells (BMSCs)-Derived miR-31 in the Pathogenesis of Recurrent Abortion by Regulating Kisspeptins 1 (KISS1) Expression | Recurrent Abortion | 4 |
| Human Endometrial Stromal Cell Differentiation is Stimulated by PPARβ/δ Activation: New Targets for Infertility? | human endometrial cell differentiation | 4 |
| Bone marrow mesenchymal stem cells combined with estrogen synergistically promote endometrial regeneration and reverse EMT via Wnt/β-catenin signaling pathway | Intrauterine adhesion | 4 |
| Adipose tissue-derived regenerative cells improve implantation of fertilized eggs in thin endometrium | thin endometrium | 4 |
| Recovery of ovarian function by human embryonic stem cell-derived mesenchymal stem cells in cisplatin-induced premature ovarian failure in mice | Premature Ovarian Failure | 4 |
| Clinical analysis of human umbilical cord mesenchymal stem cell allotransplantation in patients with premature ovarian insufficiency | Premature ovarian insufficiency | 5 |
| Restoring Ovarian Function With Human Placenta-Derived Mesenchymal Stem Cells in Autoimmune-Induced Premature Ovarian Failure Mice Mediated by Treg Cells and Associated Cytokines | Premature Ovarian Failure | 4 |
| Bone marrow-derived cells or C-X-C motif chemokine 12 (CXCL12) treatment improve thin endometrium in a mouse model | thin endometrium | 4 |
| Interceed combined with bone marrow mesenchymal stem cells improves endometrial receptivity of intrauterine adhesion | Intrauterine adhesion | 4 |
| Transplantation of umbilical cord-derived mesenchymal stem cells on a collagen scaffold improves ovarian function in a premature ovarian failure model of mice | Premature Ovarian Failure | 4 |
| Protective properties of heme oxygenase-1 expressed in umbilical cord mesenchymal stem cells help restore the ovarian function of premature ovarian failure mice through activating the JNK/Bcl-2 signal pathway-regulated autophagy and upregulating the circulating of CD8CD28 ...+- | Premature Ovarian Failure | 4 |
| Endometrial mesenchymal stem cells isolated from menstrual blood repaired epirubicin-induced damage to human ovarian granulosa cells by inhibiting the expression of Gadd45b in cell cycle pathway | epirubicin-induced damage to human ovarian granulosa cells (GCs) | 4 |
| Bone marrow mesenchymal stem cell-derived exosomal miR-144-5p improves rat ovarian function after chemotherapy-induced ovarian failure by targeting PTEN | chemotherapy-induced ovarian failure | 4 |
| Vitamin C plus hydrogel facilitates bone marrow stromal cell-mediated endometrium regeneration in rats | Intrauterine adhesion | 4 |
| Hypoxia-sensitive miRNA regulation via CRISPR/dCas9 loaded in hybrid exosomes: A novel strategy to improve embryo implantation and prevent placental insufficiency during pregnancy | endometrial receptivity and implantation success rate | 4 |
| Autocross-linked hyaluronic acid gel and adipose-derived mesenchymal stem cell composites for the treatment intrauterine adhesions | Intrauterine adhesion | 4 |
| Injectable Mesenchymal Stem Cell-Laden Matrigel Microspheres for Endometrium Repair and Regeneration | Endometrial injury and intrauterine adhesions | 4 |
| Protective effect of human umbilical cord mesenchymal stem cell exosomes on preserving the morphology and angiogenesis of placenta in rats with preeclampsia | Preeclampsia | 4 |
| Apoptotic bodies of bone marrow mesenchymal stem cells inhibit endometrial stromal cell fibrosis by mediating the Wnt/8-catenin signaling pathway | Intrauterine adhesion | 4 |
| hUMSCs restore ovarian function in POI mice by regulating GSK3β-mediated mitochondrial dynamic imbalances in theca cells | Premature ovarian insufficiency | 4 |
| Decidual stromal cell-derived exosomes deliver miR-22-5p_R-1 to suppress trophoblast metabolic switching from mitochondrial respiration to glycolysis by targeting PDK4 in unexplained recurrent spontaneous abortion | Unexplained recurrent spontaneous abortion | 2 |
| Intraovarian Transplantation of Female Germline Stem Cells Rescue Ovarian Function in Chemotherapy-Injured Ovaries | Chemotherapy-Injured Ovaries | 4 |
| Amniotic Fluid Stem Cells Prevent Follicle Atresia and Rescue Fertility of Mice with Premature Ovarian Failure Induced by Chemotherapy | Premature Ovarian Failure | 4 |
| Exosomal miR-10a derived from amniotic fluid stem cells preserves ovarian follicles after chemotherapy | Chemotherapy (CTx)-induced premature ovarian failure (POF) | 4 |
| Mesenchymal stem cells-derived exosomes improve pregnancy outcome through inducing maternal tolerance to the allogeneic fetus in abortion-prone mating mouse | Recurrent pregnancy loss (RPL) | 4 |
| Heme oxygenase-1 gene modified human placental mesenchymal stem cells promote placental angiogenesis and spiral artery remodeling by improving the balance of angiogenic factors in vitro | Abnormal placental vascular development | 4 |
| Combination Therapy of Bone Marrow Mesenchymal Stem Cell Transplantation and Electroacupuncture for the Repair of Intrauterine Adhesions in Rats: Mechanisms and Functional Recovery | Intrauterine adhesion | 4 |
| Mesenchymal Stem Cell Therapy Using Human Umbilical Cord in a Rat Model of Autoimmune-Induced Premature Ovarian Failure | Premature Ovarian Failure | 4 |
| Study of the reparative effects of menstrual-derived stem cells on premature ovarian failure in mice | Premature Ovarian Failure | 4 |
| Therapeutic Effect of Platelet-Rich Fibrin Transplant on Formation of Thin Endometrium | thin endometrium | 4 |
| Regenerative Potential of Menstrual Blood-Derived Stem Cells and Platelet-Derived Growth Factor in Endometrial Injury | Endometrial injury | 4 |
| Investigation of platelet-rich plasma in increasing proliferation and migration of endometrial mesenchymal stem cells and improving pregnancy outcome of patients with thin endometrium | thin endometrium | 5 |
| Human Acellular Amniotic Matrix with Previously Seeded Umbilical Cord Mesenchymal Stem Cells Restores Endometrial Function in a Rat Model of Injury | Endometrial injury | 4 |
| The Therapeutic Potential of Umbilical Cord Mesenchymal Stem Cells in Mice Premature Ovarian Failure | Premature Ovarian Failure | 4 |
| Effect of Human Umbilical Cord Mesenchymal Stem Cell Transplantation in a Rat Model of Preeclampsia | Preeclampsia | 4 |
| Activated Human Umbilical Cord Blood Platelet-Rich Plasma Enhances the Beneficial Effects of Human Umbilical Cord Mesenchymal Stem Cells in Chemotherapy-Induced POF Rats | Premature Ovarian Failure | 4 |
| Human amniotic epithelial cells can differentiate into granulosa cells and restore folliculogenesis in a mouse model of chemotherapy-induced premature ovarian failure | chemotherapy-induced premature ovarian failure | 4 |
| Endometriosis Impairs Bone Marrow-Derived Stem Cell Recruitment to the Uterus Whereas Bazedoxifene Treatment Leads to Endometriosis Regression and Improved Uterine Stem Cell Engraftment | Endometriosis | 4 |
| Aspirin-Mediated Reset of Preeclamptic Placental Stem Cell Transcriptome - Implication for Stabilized Placental Function | Preeclampsia | 4 |
| Human Mesenchymal Stem Cells Partially Reverse Infertility in Chemotherapy-Induced Ovarian Failure | Premature Ovarian Failure | 4 |
| Umbilical Cord Blood Mesenchymal Stem Cells as an Infertility Treatment for Chemotherapy Induced Premature Ovarian Insufficiency | Chemotherapy Induced Premature Ovarian Insufficiency | 4 |
| Transplantation of ovarian granulosa-like cells derived from human induced pluripotent stem cells for the treatment of murine premature ovarian failure | Premature Ovarian Failure | 4 |
| Transplantation of Human Menstrual Blood Stem Cells to Treat Premature Ovarian Failure in Mouse Model | Premature Ovarian Failure | 4 |
| Human amniotic mesenchymal stem cells improve the follicular microenvironment to recover ovarian function in premature ovarian failure mice | Premature Ovarian Failure | 4 |
| Small extracellular vesicles derived from embryonic stem cells restore ovarian function of premature ovarian failure through PI3K/AKT signaling pathway | Premature Ovarian Failure | 4 |
| Mesenchymal Stem Cells Ameliorate Th1-Induced Pre-Eclampsia-Like Symptoms in Mice via the Suppression of TNF-α Expression | Preeclampsia | 4 |
| ExosomalmicroRNA-139-5p from mesenchymal stem cells accelerates trophoblast cell invasion and migration by motivation of theERK/MMP-2 pathway via downregulation ofprotein tyrosine phosphatase | Preeclampsia | 4 |
| Homing and Restorative Effects of Bone Marrow-Derived Mesenchymal Stem Cells on Cisplatin Injured Ovaries in Rats | Premature Ovarian Failure | 4 |
| Hyaluronic Acid Hydrogel Integrated with Mesenchymal Stem Cell-Secretome to Treat Endometrial Injury in a Rat Model of Asherman's Syndrome | Asherman's syndrome | 4 |
| Adoptive transfers of CD4CD25Tregs partially alleviate mouse premature ovarian insufficiency++ | Premature ovarian insufficiency | 4 |
| Sodium alginate-bioglass-encapsulated hAECs restore ovarian function in premature ovarian failure by stimulating angiogenic factor secretion | Premature Ovarian Failure | 4 |
| Synergistic regenerative therapy of thin endometrium by human placenta-derived mesenchymal stem cells encapsulated within hyaluronic acid hydrogels | thin endometrium | 4 |
